# Chemical fingerprinting of a furniture waste landfill fire: parent and alkylated PAHs, organophosphates and environmental-health risk assessments

**DOI:** 10.1007/s10653-026-03555-4

**Published:** 2026-10-23

**Authors:** Wojciech Rykała, Monika J. Fabiańska, Dominika Dąbrowska

**Affiliations:** Institute of Earth Sciences, Faculty of Natural Sciences, University of Silesia in Katowice, Będzińska 60, 41-200 Sosnowiec, Poland

**Keywords:** Municipal landfill, Furniture waste fire, Parent and alkylated PAHs, Organophosphate esters (OPEs), Diagnostic ratios, Environmental and health indicators

## Abstract

Fires at furniture-waste storage sites are an under-researched source of persistent organic contaminants. This study characterized 13 charred furniture-waste samples collected after a landfill fire in Tychy, Poland, using GC–MS to determine parent and alkylated PAHs and organophosphate esters (OPEs), and to perform screening-level environmental and human-health assessments. The sum of the PAHs ranged from 34.56 to 3031.86 µg/g, with the highest burden in TM7; the maximum benzo(a)pyrene-equivalent concentration was 155.31 µg/g. The thermal fingerprint of the fire was reflected by the dominance of 5-ring PAHs in the most intensely burned material and by altered alkylated-PAH ratios in the fire core. TCiPP, TPhP and TCP were detected in all samples, with TPhP reaching 43.97 mg/g. Furthermore, the systematic identification of abietane derivatives, including dehydroabietic acid, aldehyde, and methyl ester, verified the extensive thermal transformation of coniferous softwood matrices within the burned furniture materials. Under the equilibrium, no-attenuation water-phase screening scenario, source-zone pore-water BaP-equivalent concentrations ranged from 0.24 to 5.53 µg/L and calculated cancer-risk estimates ranged from 2.78×10^−6^ to 6.50×10⁻^5^. Under the upper-bound direct-contact scenario (100 mg/day incidental soil-plus-dust ingestion), total cancer risk ranged from 9.20×10^−7^ to 1.39×10^−4^, with TM7 exceeding 1×10^−4^. Ecological risk quotients for BaP ranged from 1.21×10^3^ to 4.73×10^4^. The results identify post-fire furniture waste as a priority source for containment and monitoring; water-phase estimates are conservative screening outputs and require confirmation by leachate and groundwater measurements.

## Introduction

Municipal and industrial waste management represents one of the modern world's greatest environmental challenges. Despite increasing emphasis on implementing the circular economy concept and the tightening of legal regulations, landfilling remains a widely used method of waste disposal in many regions of the world (Parvin & Tareq, 2021). In the European Union, notwithstanding a significant decline in the volume of waste sent to landfills—from 173 million tonnes in 2010 to 137 million tonnes in 2022—approximately 22.4% of generated municipal waste still ends up in landfills (European Environment Agency (EEA) 2026). Bulky waste (including furniture) and plastic waste pose a particular problem, as their specific chemical composition and physical properties result in long-term impacts on soil and water environments (Yingjie et al., 2026).

Furniture waste accounts for a significant share of the total volume of solid municipal waste. It is estimated that approximately 45% of furniture waste in the European Union is sent to landfills (Liu et al., 2010). In Poland, where the furniture industry plays a vital economic role, millions of tonnes of waste comprising wood-based panels and old furniture are generated annually; a substantial portion of this waste ends up in landfills due to technological and administrative barriers to recycling (Ciuła et al., 2023).

The material complexity of furniture—comprising wood, particleboard, flexible polyurethane foams, textiles, and lacquer coatings—makes it a source of numerous hazardous substances (Tahir et al., 2023) of particular concern is the presence of flame retardants, including organophosphates (OPEs), which are widely used as additives in foams and upholstery fabrics (Stapleton et al., 2009). These compounds are not permanently bound to the polymer matrix (they are so-called additive-type agents), causing them to leach relatively quickly under landfill conditions (Stubbings & Harrad, 2018). It should also be noted that furniture manufacturers often burn wood waste generated during production processes in small industrial furnaces, using it as an additional heat source (Szczurek et al., 2021). However, the combustion of these materials can generate sulfur and nitrogen compounds or volatile organic compounds (VOCs)—such as toluene, xylene, and benzene—due to the presence of waxes and paints (Escalante et al., 2022). Disposing of furniture waste in landfills is inappropriate due to its low biodegradability and the risk of hazardous chemicals leaching into the soil and groundwater (Liu et al., 2010).

At the same time, plastic waste—including packaging films—accounts for a significant portion of the mass deposited in landfills. In 2022, over 30% of plastic waste in the EU was sent to landfills (Baran, 2022). Due to their structure and often high levels of organic contamination, plastic films are difficult to recycle, resulting in their disposal in large quantities (Horodytska et al., 2018). The degradation of these materials under landfill conditions, driven by physicochemical and biological factors, leads to fragmentation and the release of microplastics (Hussain et al., 2026). Furthermore, this process involves the release of a wide range of chemical additives—such as plasticizers (e.g., phthalates, bisphenol A) and stabilizers—which leach into landfill leachate, thereby increasing its toxicity (Wojnowska-Baryła et al., 2022).

Monitoring alkylated PAH derivatives—specifically methylnaphthalenes, methylphenanthrenes, and dimethylphenanthrenes—represents an advanced approach in monitoring municipal solid waste landfill fires (Wang & Stout, 2007). In the process of thermal degradation of complex solid waste, such as furniture fractions with a high content of polymers and adhesives, these specific alkylated structures serve as excellent diagnostic indicators (Vicente et al., 2016). Their relative abundance and molecular distribution are determined by the unique pyrolysis conditions accompanying landfill fires, as well as the thermodynamic stability of individual isomers at varying combustion temperatures (Wang & Stout, 2007). Incorporating these alkylated variants into analytical procedures enables the creation of a characteristic chemical profile. This profile is of key importance for identifying source materials and reconstructing precise combustion pathways typical of complex, contemporary municipal waste heaps.

Landfill leachate is a key vector for the transport of pollutants from waste disposal sites into the environment (Saha et al., 2025). This forms the basis for implementing environmental risk assessment regarding groundwater quality in the vicinity of waste landfills (Rykała & Dąbrowska, 2020). It constitutes a highly toxic mixture of dissolved organic matter, xenobiotic compounds, heavy metals, and specific organic pollutants (Abdel-Shafy et al., 2024). In the case of unlined landfills or those with compromised isolation barriers, this leachate can freely infiltrate the aeration zone, reaching the groundwater and causing its long-term degradation (Abiriga et al., 2020).

Among the organic contaminants migrating from landfills, scientific literature pays particular attention to polycyclic aromatic hydrocarbons (PAHs) and the aforementioned organophosphate esters (OPEs). PAHs in landfills originate primarily from the slow decomposition of organic matter and pyrolytic processes occurring in the deeper layers of the waste mass, as well as from deposited ash and slag (Zakaria et al., 2005). Due to their low water solubility, PAHs exhibit a strong affinity for soil organic matter and sediments (Patel et al., 2020), and their migration into groundwater poses a serious threat given their mutagenic, carcinogenic, and teratogenic effects (Duan et al., 2023).

Meanwhile, organophosphates (OPEs)—widely used as replacements for the phased-out polybrominated diphenyl ethers (PBDEs)—are increasingly being detected in landfill leachates at very high concentrations (Qi et al., 2019). Among the most frequently detected are chlorinated esters, such as tris(2-chloroethyl) phosphate (TCEP), tris(1-chloro-2-propyl) phosphate (TCIPP), and tris(1,3-dichloro-2-propyl) phosphate (TDCIPP) (Vykoukalová et al., 2017). These compounds, released primarily from discarded furniture (PUF foam) and electronic waste, exhibit higher water solubility than PAHs, facilitating their migration via leachate into groundwater and surface water (Zhu et al., 2023). Toxicological studies indicate that OPEs have adverse effects on the reproductive system and neurological development. Furthermore, some of these compounds, such as TCEP and TDCIPP, are classified as potential carcinogens (Doherty et al., 2019; Zhang et al., 2024).

The aims of this research was: (1) to apply diagnostic ratios of parent and alkylated polycyclic aromatic hydrocarbons (PAHs) as a chemical fingerprint to identify the source materials involved in the fire; (2) assess the environmental and human health impacts of the fire incident at the municipal waste landfill in the Tychy (Southern Poland), which stores significant quantities of furniture waste; (3) to understand the combustion processes during the furniture waste fire and characterize their effect on fire spread under in-situ conditions.

It is crucial to highlight that field studies investigating this specific category of solid waste under real, *in-situ* conditions remain unprecedented. This novel approach provides critical insights and practical tools for analyzing the dynamic behavior of furniture waste combustion processes. Furthermore, the environmental impact of municipal waste landfill fires is poorly recognized in literature and incidents themselves often overlooked since they are erroneously considered accidental and scarce. Thus, understanding the mechanisms governing the release and migration of specific emerging contaminants—such as PAHs and organophosphate esters (OPEs)—from landfilled furniture waste fractions is essential for comprehensively assessing both short- and long-term environmental and public health risks.

## Materials and methods

### Study area

**Fig. 1:**
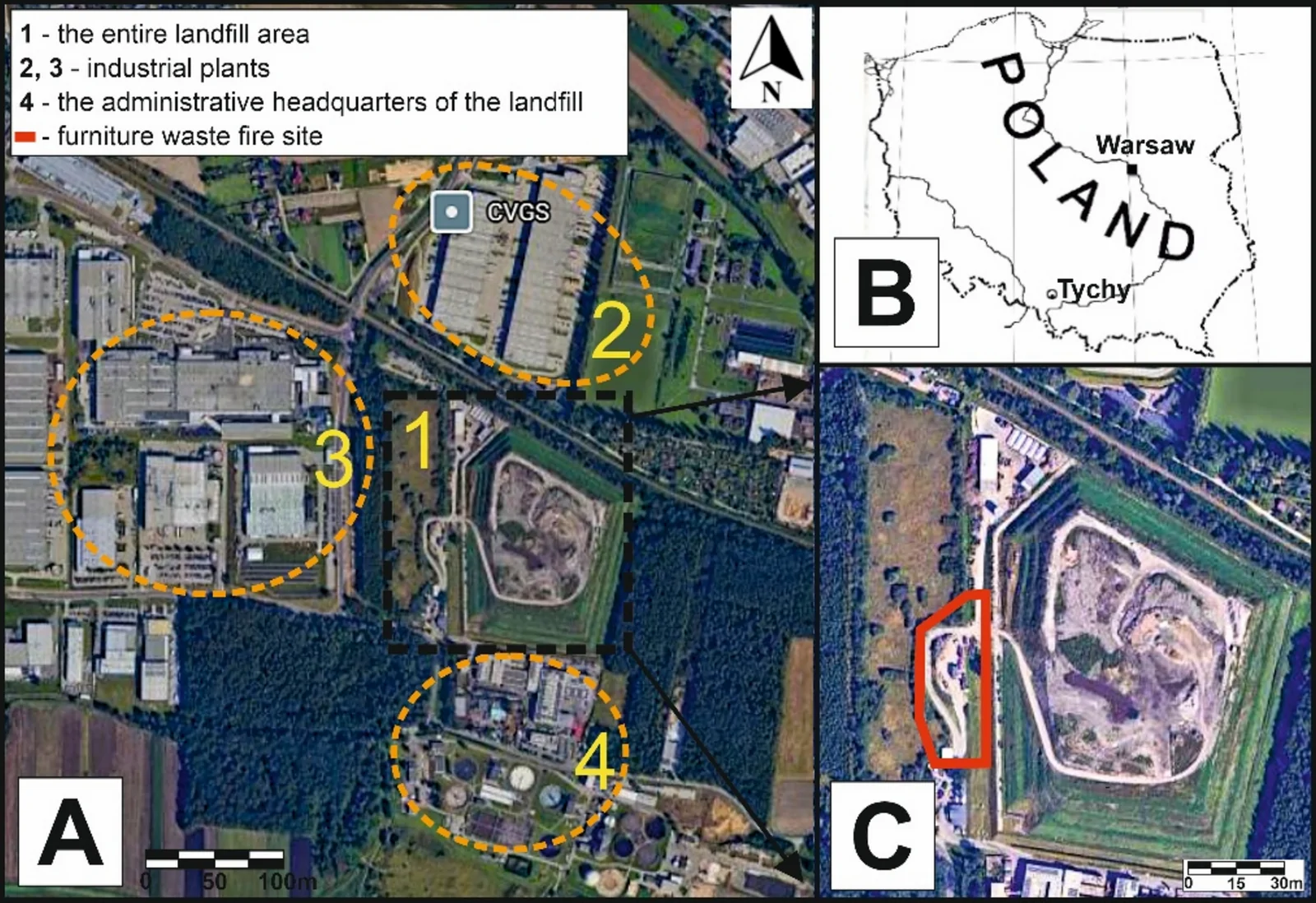
**A**–**C** Location of the research

The municipal waste landfill in the Tychy was established in 1994 (Cell I), covering an area of 3.5 hectares, and operated until 2004. It was closed due to the lack of adequate safeguards to prevent the migration of contaminants into the soil (Dąbrowska & Witkowski, 2022). Subsequently, in the same year, the currently active landfill cell (Cell II) and its associated infrastructure were put into operation; the facility covers an area of 12.7 hectares. Situated between these cells is an area designated for the receiving, sorting, and processing of furniture waste, where an uncontrolled fire involving such waste occurred (Fig. 1)—covering an area of approximately 1 hectare.

A fire broke out at a furniture waste storage site on August 19, 2025, at 4:00 a.m. Because the waste was piled up to heights of six meters, the fire spread rapidly, eventually engulfing an area of approximately 1000 square meters. It generated heavy smoke, with black plumes visible from many kilometers away. Seven fire crews responded to the incident, and the fire was finally extinguished after 6:00 a.m. No one was injured. The likely cause of the fire was identified as biochemical processes leading to spontaneous combustion within the deep layers of the waste pile. At the time, weather conditions were stable, dry, and calm, with temperatures around 10 °C. The fire produced a heavy smoke cloud that settled low to the ground and drifted over nearby areas, directly affecting the level of risk to the local population.

### Sampling and solvent extraction

**Fig. 2:**
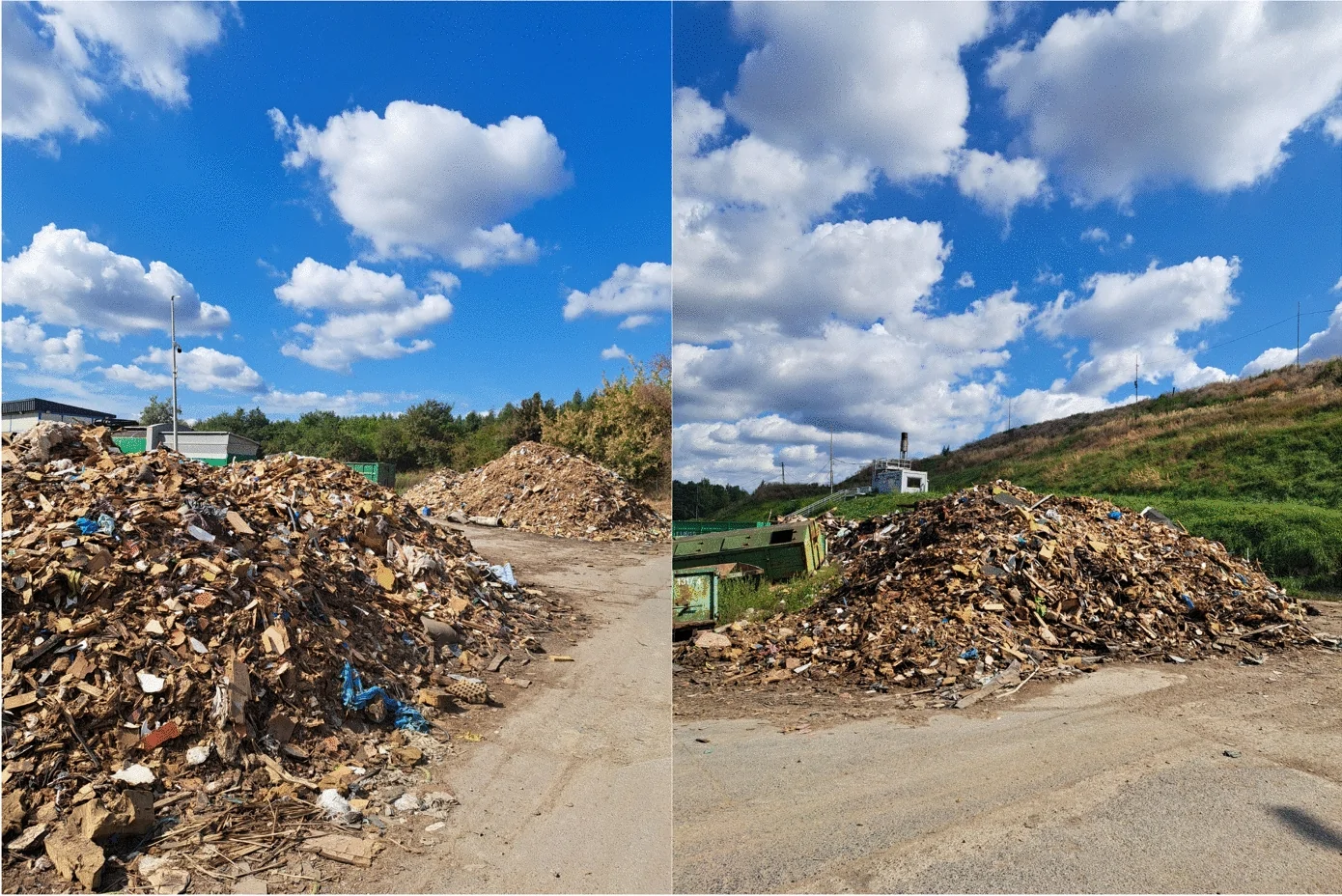
The site of the burnt furniture waste landfill in Tychy (August 2025)

**Fig. 3:**
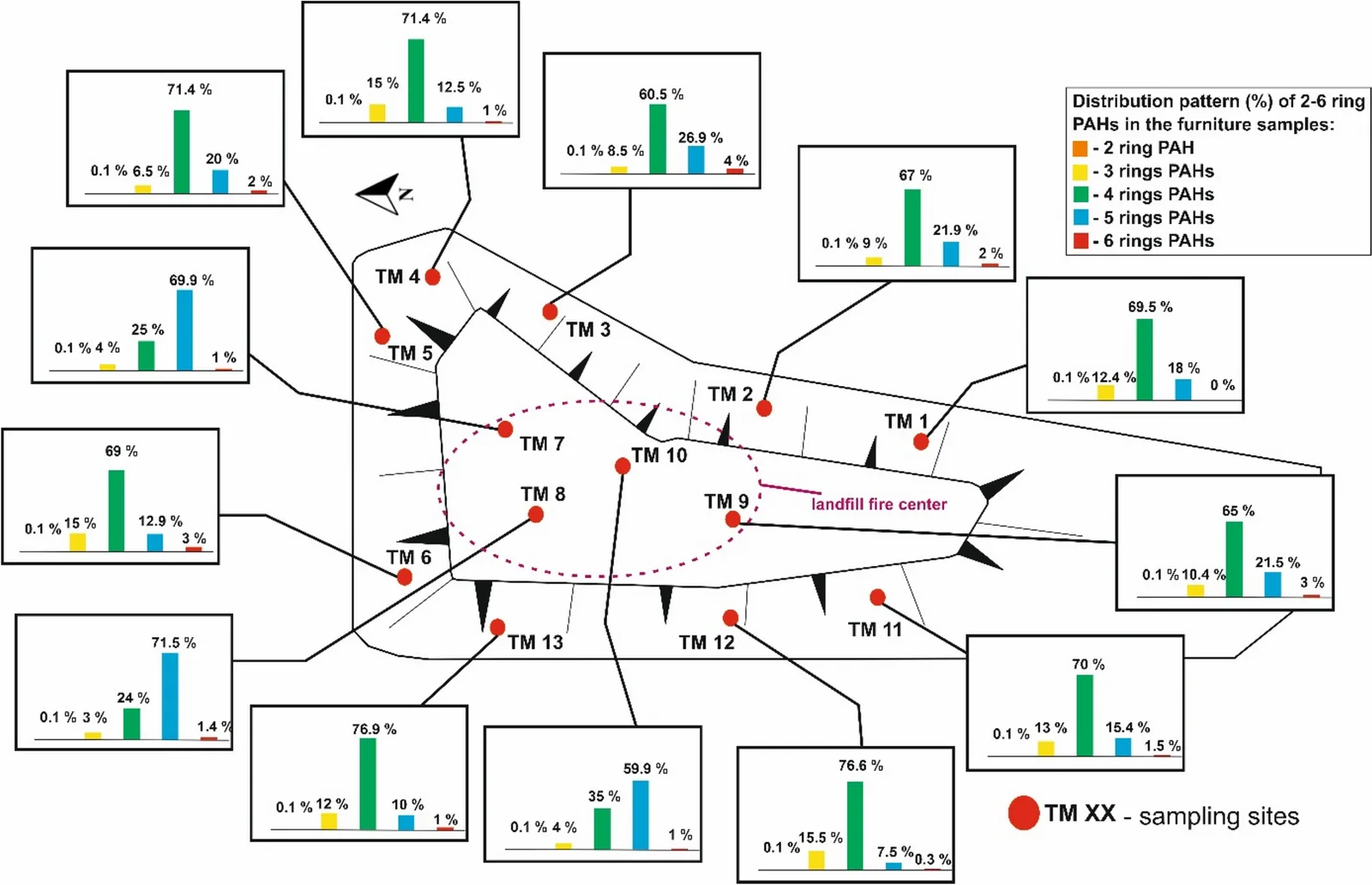
The location of furniture waste sampling sites and distribution (%) of PAHs

Laboratory testing specimens were gathered across the landfill site (Fig. 2) by following a predetermined route. During a field inspection on August 25, 2025, a total of 13 samples of charred furniture waste were collected and coded as TM1 through TM13 (Fig. 3). Post-fire sampling covered both the core storage area and the facility's perimeter. The incinerated waste items were sampled at intervals ranging from 1 to 3 m. The objective of this procedure was to evaluate the pollutant formation dynamics and their distribution patterns following deposition. Consequently, the site visit was executed a few days after the fire, as soon as access to the facility was officially permitted.

For analytical purposes, approximately 20 g portions of the charred furniture residues were weighed out. The collected material was sealed in airtight glass jars and transferred to the laboratory facility. To achieve an air-dried state, the specimens were subjected to ambient desiccation at room temperature (approx. 22 °C) for roughly 5 days under sterile, monitored laboratory conditions. Once appropriately dried and cataloged, the samples underwent extraction with dichloromethane (DCM) inside an ultrasonic bath for 15 min at a constant temperature of 30 °C. The resulting extracts were filtered through paper discs (grade 3 m/N; 110 mm diameter), followed by solvent evaporation to determine the final extraction yield. The definitive phase involved analyzing the obtained extracts via gas chromatography-mass spectrometry (GC–MS).

### Gas chromatography-mass spectrometry (GC–MS)

Chromatographic analyses of the samples were performed at the Faculty of Natural Sciences of the University of Silesia in Katowice, Poland. The instrumentation consisted of an Agilent Technologies 7890A gas chromatograph coupled with an Agilent 5975C mass spectrometer equipped with a triple-axis detector. Helium (6.0 Grade) served as the carrier gas, maintained at a constant flow rate of 2.6 mL/min. Compound separation was carried out on a J&W HP5-MS fused silica capillary column (60 m × 0.32 mm i.d., 0.25 μm film thickness) featuring a chemically bonded 5% phenyl, 95% methylsiloxane stationary phase. The GC oven temperature program was initialized at 45 °C (held for 1 min), increased to 100 °C at a rate of 20 °C/min, and subsequently raised to 300 °C at 3 °C/min, with a final hold time of 60 min. A solvent delay of 10 min was applied. Mass spectra were acquired in electron impact (EI) mode at an ionization energy of 70 eV using full-scan monitoring. The monitored mass ranges were set from 45 to 550 Da for the first 40 min of the run, and from 50 to 700 Da for retention times exceeding 40 min. Data processing was conducted via Hewlett-Packard ChemStation software. The components were identified by evaluating their mass spectra, comparing their retention times with reference standards, interpreting MS fragmentation pathways, and cross-referencing with published literature (Philp, 1985).

The peak areas for PAHs were integrated manually. Quantification was executed using five-point calibration curves constructed from analytical standards. Linearity between the chromatographic responses and PAH concentrations was validated across a range of 0.10–10 μg/mL, yielding correlation coefficients between 0.997 and 0.998. To verify method validity, the NIST SRM 1649b standard reference material was analysed, and the recovered values were evaluated against the certified PAH concentrations. Limits of detection (LODs) were established based on a threefold standard deviation of the background noise determined from triplicate analyses of procedural blanks. The mean LOD was calculated to be 2.0 ± 0.05 ng/mL. For data processing, any concentration falling below the LOD threshold was designated as zero. As part of the quality assurance and quality control (QA/QC) protocol, a procedural blank was processed alongside every sample batch to monitor and account for potential background contamination.

Chromatographic peaks for organophosphate esters (OPEs) were integrated manually. Analytical standards including TPhP, TCEP, TBEP, and TCiPP (as an isomeric mixture) were sourced from Sigma-Aldrich, alongside their synthesis-grade reagents TCP, TBP, and 99% pure TEP. Prior to establishing the calibration curves, the purity of each reference compound was verified under the exact analytical parameters employed for the sample extracts. Linear regressions between peak areas and OPE concentrations were validated within a 0.10–10 μg/mL range, yielding correlation coefficients of 0.997–0.998. To adhere to quality assurance and quality control (QA/QC) guidelines, a procedural blank was incorporated into each analytical batch to track potential secondary contamination. Reagent contamination was ruled out, as no target analytes were detected in the blank matrices. The instrumental limits of detection (LOD) were determined based on a minimum signal-to-noise ratio of three (S/N ≥ 3), resulting in an average LOD of 3.5 ± 0.05 ng/mL. For subsequent statistical evaluations, any concentration below the LOD threshold was assigned a value of zero.

### Screening-level environmental-health risk assessment

The groundwater-ingestion assessment was conducted as a screening-level Human Health Risk Assessment (HHRA) for adult residents. Input data were direct measurements of PAH concentrations in solid charred furniture waste samples (TM1–TM13), expressed in µg/kg. Because groundwater and leachate were not sampled, the water-phase calculation was not used to predict a measured or site-specific aquifer concentration (EPA, 1996).

Equation (1) estimates a screening-level source-zone pore-water concentration (C_pw_) under an assumed local equilibrium between the solid phase and infiltrating water. The soil parameters were retained as conservative surrogate assumptions in the absence of waste-specific hydraulic and sorption measurements.

1 $$C_{pw} = C_{s} /\left[ {\left( {Koc \, * \, foc} \right) + \left( {\Theta_{w} /\rho_{b} } \right)} \right]$$

where:

C_pw_—screening-level concentration in source-zone pore water (µg/L);

C_s_—the measured concentration of the specific PAH in the solid waste sample (µg/kg);

Koc—the organic carbon–water partition coefficient (L/kg), specific to each PAH;

foc—the fraction of organic carbon in the soil, conservatively assumed as 0.006 (0.6%) for sandy soils;

Θ_w_—the volumetric water content (0.30 L/L);

ρ_b_—the soil bulk density (1.70 kg/L).

This calculation assumes homogeneous material, linear equilibrium sorption and direct solid–water contact, and it does not account for desorption kinetics, unsaturated-zone thickness, infiltration, dissolved organic matter, dilution, retardation or degradation during transport. Where a groundwater screening concentration is required, C_gw,screen_ = C_pw_/DAF; DAF = 1 was used as a deliberately conservative no-dilution/no-attenuation case. Thus, the water-phase results are screening estimates that require leachate and groundwater monitoring for site-specific confirmation.

The water-ingestion scenario evaluated hypothetical ingestion of water with the screening concentration C_gw,screen_. The Average Daily Dose (ADD) for non-carcinogenic effects and the Lifetime Average Daily Dose (LADD) for carcinogenic effects, both expressed in mg/kg/day, were calculated using standard US EPA equations:

2 $${\mathrm{ADD}} = \left( {{\mathrm{C}}_{{{\mathrm{gw}}}} *{\mathrm{IR}}*{\mathrm{EF}}*{\mathrm{ED}}} \right)/\left( {{\mathrm{BW}}*{\mathrm{AT}}_{{\mathrm{n}}} *1000} \right)$$

3 $${\mathrm{LADD}} = \left( {{\mathrm{C}}_{{{\mathrm{gw}}}} *{\mathrm{IR}}*{\mathrm{EF}}*{\mathrm{ED}}} \right)/\left( {{\mathrm{BW}}*{\mathrm{AT}}_{{\mathrm{c}}} *1000} \right)$$

The exposure parameters were adopted from the US EPA Exposure Factors Handbook (US EPA, 2011):

IR (Ingestion Rate)—2.0 L/day;

EF (Exposure Frequency)—350 days/year;

ED (Exposure Duration)—30 years;

BW (Body Weight)—70 kg;

AT_n_ (Averaging Time for non-carcinogens)—ED * 365 days;

AT_c_ (Averaging Time for carcinogens)—70 * 365 days.

To evaluate the carcinogenic risk of the PAH mixture, the Relative Potency Factor (RPF) approach was utilized. This method converts the concentration of individual carcinogenic PAHs into benzo(a)pyrene equivalents (BaPeq) based on their relative toxicity compared to benzo(a)pyrene (BaP) (EPA, 1993). The total carcinogenic risk (CR) was calculated by multiplying the LADD of BaPeq by the oral Cancer Slope Factor (CSF) for BaP, which is 1.0 (mg/kg/day)^−1^ according to the US EPA Integrated Risk Information System (IRIS).

4 $${\mathrm{CR}} = \sum \left( {{\mathrm{LADD}}\_{\mathrm{i}}*{\mathrm{RPF}}\_{\mathrm{i}}} \right)*{\mathrm{CSF}}\_{\mathrm{BaP}}$$

For non-carcinogenic risk, the Hazard Quotient (HQ) was calculated by dividing the ADD by the specific Reference Dose (RfD) for each compound. The overall non-carcinogenic risk was expressed as the Hazard Index (HI), which is the sum of individual HQs (Dąbrowska & Witkowski, 2022).

According to US EPA guidelines, a CR value below 10^–6^ is considered acceptable, values between 10^–6^ and 10^–4^ indicate an elevated risk requiring attention, and values exceeding 10^–4^ are deemed unacceptable. For non-carcinogenic effects, an HI value below 1 indicates no significant risk of adverse health effects.

A direct-contact screening assessment was also conducted for contaminated solid furniture waste. The scenario represents incidental contact with soil and dust derived from weathered surface waste, rather than measured ingestion of bulk charred furniture waste. Three pathways were evaluated for an adult receptor: incidental soil/dust ingestion, dermal contact and inhalation of resuspended dust. BaP-equivalent concentrations were calculated for each sample using the RPF-weighted sum of individual carcinogenic PAHs (EPA, 1993).

The Lifetime Average Daily Dose (LADD) for carcinogenic effects was calculated using standard US EPA soil-exposure equations (EPA, 1989, 2011). The direct-contact assessment is scenario-based and does not estimate a measured individual intake. The general exposure parameters (EF, ED, BW and AT_c_) are those stated above; the uncertainty in the ingestion term was examined by sensitivity analysis.

5 $${\mathrm{LADD}}_{{{\mathrm{\_ing}}}} = \left( {{\mathrm{C}}_{{\mathrm{s}}} \times {\mathrm{IR}}_{{\mathrm{s}}} \times {\mathrm{CF}} \times {\mathrm{EF}} \times {\mathrm{ED}}} \right)/\left( {{\mathrm{BW}} \times {\mathrm{AT}}_{c} } \right)$$

6 $${\mathrm{LADD}}_{{{\mathrm{\_derm}}}} = \left( {{\mathrm{C}}_{{\mathrm{s}}} \times {\mathrm{SA}} \times {\mathrm{AF}} \times {\mathrm{ABS}} \times {\mathrm{CF}} \times {\mathrm{EF}} \times {\mathrm{ED}}} \right)/\left( {{\mathrm{BW}} \times {\mathrm{AT}}_{c} } \right)$$

7 $${\mathrm{LADD}}_{{{\mathrm{\_inh}}}} = \left( {{\mathrm{C}}_{{\mathrm{s}}} \times \left( {1/{\mathrm{PEF}}} \right) \times {\mathrm{IR}}_{{\mathrm{a}}} \times {\mathrm{EF}} \times {\mathrm{ED}}} \right)/\left( {{\mathrm{BW}} \times {\mathrm{AT}}_{c} } \right)$$

where (soil-specific parameters):

C_s_–concentration of benzo(a)pyrene equivalents (BaP) in the solid waste (mg/kg);

IR_s_–incidental soil/dust ingestion rate. Values of 10 mg/day (central tendency for soil only), 50 mg/day (upper-percentile soil only) and 100 mg/day (upper-percentile soil plus dust) were evaluated; 100 mg/day is retained only as an upper-bound sensitivity case;

CF–unit conversion factor: 10^−6^ kg/mg;

SA–skin surface area available for contact: 5700 cm^2^/day;

AF–soil-to-skin adherence factor: 0.07 mg/cm^2^;

ABS–dermal absorption fraction for PAHs: 0.13;

PEF–particulate emission factor: 1.36 × 10^9^ m^3^/kg;

IR_a_–inhalation rate: 20 m^3^/day.

The total carcinogenic risk (CR) was calculated as the sum of risks from all three pathways, using the oral and dermal Cancer Slope Factor (CSF) of 1.0 (mg/kg·day)⁻^1^ for BaP (ECHA, 2012), and an inhalation CSF of 2.1 (mg/kg·day)⁻^1^ derived from the Inhalation Unit Risk (IUR = 0.0006 (µg/m^3^)⁻^1^):

8 $${\mathrm{CR}}_{{{\mathrm{\_total}}}} = \left( {{\mathrm{LADD}}_{{{\mathrm{\_ing}}}} \times {\mathrm{CSF}}_{{\mathrm{o}}} } \right) + \left( {{\mathrm{LADD}}_{{{\mathrm{\_derm}}}} \times {\mathrm{CSF}}_{{\mathrm{d}}} } \right) + \left( {{\mathrm{LADD}}_{{{\mathrm{\_inh}}}} \times {\mathrm{CSF}}_{{\mathrm{i}}} } \right)$$

An Ecological Risk Assessment (ERA) was performed to evaluate the potential adverse effects of the PAH contamination on terrestrial ecosystems in the vicinity of the waste deposit. The Risk Quotient (RQ) method was applied, comparing the measured concentrations of benzo(a)pyrene—the most toxic and well-characterized PAH—to its Predicted No-Effect Concentration (PNEC) for soil organisms:

9 $${\mathrm{RQ}} = {\mathrm{C}}_{{\mathrm{s}}} /{\mathrm{PNEC}}_{{{\mathrm{soil}}}}$$

The PNEC value for BaP in soil was set at 0.0006 mg/kg (dry weight), as established by the European Chemicals Agency (ECHA) based on ecotoxicological studies on soil invertebrates and plants (ECHA, 2012). This value represents the concentration below which no adverse effects on soil-dwelling organisms are expected. For comparison, the Polish regulatory soil quality standards (Ministry of the Environment, 2016) were also considered, with BaP threshold values of 0.2 mg/kg for Group I soils (agricultural, residential) and 2.0 mg/kg for Group III soils (industrial, commercial).

### Data processing, diagnostic ratios and calculated reflectance equivalent

Diagnostic ratios were calculated from manually integrated GC–MS peak areas after blank correction. The parent-PAH ratios were A/(A+P), Fl/(Fl+Py), BaA/(BaA+Ch) and IP/(IP+BghiP), where A, P, Fl, Py, BaA, Ch, IP and BghiP denote anthracene, phenanthrene, fluoranthene, pyrene, benzo[a]anthracene, chrysene, indeno[1,2,3-cd]pyrene and benzo[ghi]perylene, respectively. The alkylnaphthalene and alkylphenanthrene parameters were calculated as MNR = 2-MN/1-MN; MPI-3 = (2-MP+3-MP)/(1-MP+9-MP); MPI-1 = 1.5×(2-MP+3-MP)/(P+1-MP+9-MP); and DMPR = (2,6-DMP+2,7-DMP)/(3,10-DMP+1,9-DMP+1,6-DMP), where MN, MP and DMP denote the relevant methylated isomers (Radke et al., 1982, 1986).

A calculated reflectance equivalent was derived as R_c_ = 0.60×MPI-1+0.40. This empirical relationship was developed for sedimentary organic matter within a defined maturity range. In the present study, R_c_ is therefore used only as a comparative molecular proxy for changes in carbonized material.

## Results and discussion

### PAH concentration in incinerated waste samples

**Fig. 4:**
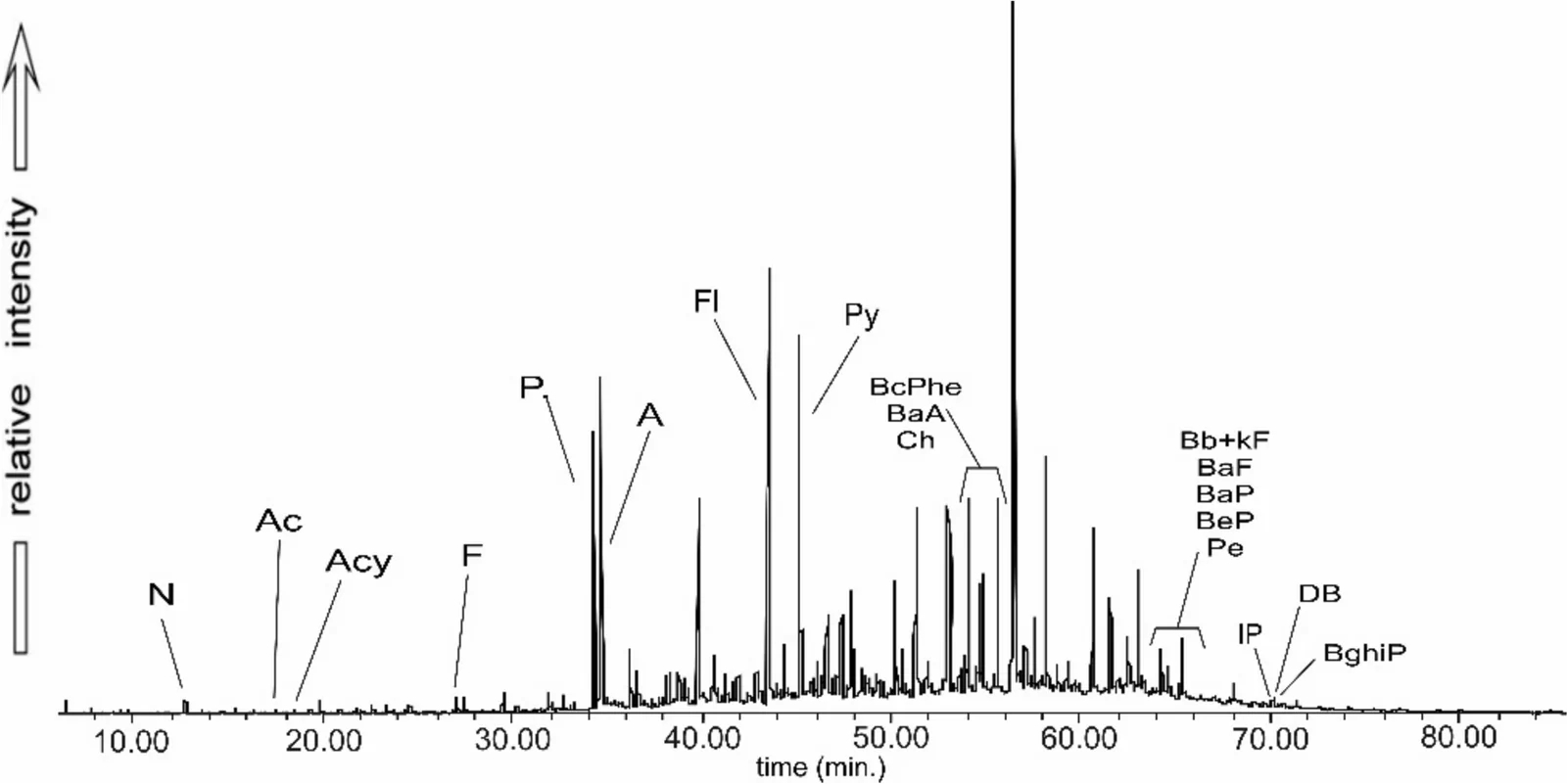
Ion chromatograms of generalized PAHs distribution in extract TM7

In the thirteen samples collected from the furniture waste landfill fire (TM1–TM13) (Table 1 and Fig. 4), twenty distinct polycyclic aromatic hydrocarbons (PAHs) were identified. The compounds included naphthalene (N, *m/z* = 128), fluorene (F, *m/z* = 166), phenanthrene (P, *m/z* = 178), anthracene (A, *m/z* = 178), acenaphthene (Ac, *m/z* = 154), and acenaphthylene (Acy, *m/z* = 152). Furthermore, the analytical screening confirmed the presence of fluoranthene (Fl, *m/z* = 202), pyrene (Py, *m/z* = 202), benzo(c)phenanthrene (BcPhe, *m/z* = 252), benzo(a)anthracene (BaA, *m/z* = 228), and chrysene (Ch, *m/z* = 228). The remaining identified PAHs consisted of benzo(b+k)fluoranthene (Bb+kF, *m/z* = 252), benzo(a)fluoranthene (BaF, *m/z* = 252), benzo(c)fluoranthene (BcF, *m/z* = 252), benzo(a)pyrene (BaP, *m/z* = 252), benzo(e)pyrene (BeP, *m/z* = 252), perylene (Pe, *m/z* = 252), dibenzo(a+h)anthracene (DB, *m/z* = 278), indeno[1,2,3-cd]pyrene (IP, *m/z* = 276), and benzo(ghi)perylene (BghiP, *m/z* = 276).

**Table 1:** Polycyclic aromatic hydrocarbon concentrations in the Tychy samples (µg/g)

PAH\Sample	TM1	TM2	TM3	TM4	TM5	TM6	TM7	TM8	TM9	TM10	TM11	TM12	TM13
N	0.011	0.006	0.001	0.039	0.010	0.023	0.019	0.006	0.017	0.028	0.009	0.027	0.009
P	3.748	3.459	4.890	8.954	4.490	5.732	71.304	5.243	3.866	22.123	3.419	18.327	12.401
A	0.354	0.423	0.242	1.244	0.569	0.753	44.431	0.671	0.460	2.393	0.466	2.577	1.819
Ac	0.121	0.105	0.343	0.256	0.158	0.165	1.627	0.195	0.250	0.418	0.114	0.175	0.189
Acy	0.174	0.196	0.129	0.674	0.331	0.488	0.886	0.315	0.327	0.493	0.271	0.600	0.702
F	0.192	0.213	0.300	0.525	0.281	0.351	2.638	0.378	0.263	0.635	0.247	0.590	0.483
Fl	10.662	12.558	17.192	24.110	25.825	13.616	308.044	18.513	12.733	99.494	10.073	47.723	43.171
Py	9.786	11.539	14.413	21.348	23.076	12.756	288.695	16.794	11.963	82.202	8.855	40.330	35.832
BaA	2.084	3.725	4.019	4.460	6.266	3.227	63.128	6.663	3.373	17.584	2.080	8.398	7.830
Ch	2.654	4.778	5.334	5.803	7.760	4.156	85.793	8.107	4.056	21.777	2.778	10.586	9.245
BcPhe	0.559	0.846	0.738	1.050	1.099	0.758	9.682	1.192	0.623	3.103	0.435	1.888	1.777
Bb+kF	2.608	4.365	7.630	3.935	8.033	2.926	66.461	9.824	4.633	19.657	2.252	4.632	5.674
BaF	0.306	0.563	0.828	0.466	0.830	0.286	5.624	1.027	0.514	1.760	0.244	0.679	0.721
BaP	0.724	1.562	2.918	1.423	2.510	0.807	28.386	3.597	1.700	7.429	0.792	1.442	2.022
BeP	2.560	4.315	6.785	3.664	6.419	2.485	50.353	8.057	3.791	15.006	1.916	3.730	4.344
Pe	0.399	0.247	0.398	0.185	0.495	0.140	5.477	0.627	0.305	1.398	0.115	0.195	0.303
DB	–	–	–	–	–	–	1965.46	129.968	–	334.416	–	–	–
IP	–	0.197	0.473	0.228	0.283	0.259	6.968	0.645	0.283	1.499	0.105	0.096	0.344
BghiP	–	0.816	2.023	0.860	1.190	1.116	26.885	2.294	1.315	5.331	0.393	0.295	1.086

N, Naphthalene, P, Phenanthrene, A, Anthracene, Ac, Acenaphthene, Acy, Acenaphthylene, F, Fluorene, Fl, Fluoranthene, Py, Pyrene, BaA, Benzo(a)anthracene, Ch, Chrysene, BcPhe, Benzo(c)phenanthrene, Bb+Kf, Benzo(b+k) fluoranthene, BaF, Benzo(a)fluoranthene, BaP, Benzo(a)pyrene, BeP, Benzo(e)pyrene, Pe, Perylene, DB, Dibenzo(a+h)anthracene, IP, Indeno[1,2,3-cd]pyrene, BghiP, Benzo(ghi)perylene, "-" Compound not found

The average total PAH content was determined to be 526.35 µg/g across 13 incinerated solid furniture waste samples. Peak concentrations were recorded within the fire's core zone, specifically in samples TM7, TM10, TM8, and TM5, yielding values of 5129.78, 831.96, 347.98, and 109.41 µg/g, respectively. To evaluate the contamination levels, PAH concentrations were classified into four distinct groups: (i) minimal (< 50 µg/g), (ii) elevated (50–100 µg/g), (iii) high (101—1000 µg/g), and (iv) extreme (> 1000 µg/g). One sample (TM7) fell into the extreme category (iv). Meanwhile, category (iii) comprised three solid waste samples (TM5, TM8, TM10). The lowest category (i) is represented by six samples (TM1, TM2, TM9, TM11-TM13).

The dominant group of PAHs (Fig. 3) consists of 4-ring compounds (including Fl, Py, BaA, Ch, BcPhe). They were predominant in 10 tested samples (TM1–TM6, TM9, TM11–TM13), accounting for 60.5% to 76.9%. Fluoranthene and pyrene recorded the highest values, which are consistent with their elevated concentrations during the combustion of furniture waste (Hoffer et al., 2020). The primary driving force behind the formation of 4-ring structures (mainly fluoranthene and pyrene) is the breakdown of lignin structures combined with the presence of synthetic furniture binders (Colom-Díaz et al., 2017). Combustion of this type of waste at temperatures between 400 °C and 600 °C leads to the dominance of 4-ring PAHs. Studies show that the emission of these compounds during the combustion of MDF boards and furniture is over a dozen times higher than in the case of clean wood (Han et al., 2020; Klyta et al., 2023). This 4-ring dominance also presents a distinct fingerprint compared to other emission sources. Unlike open tire landfill fires, which are characterized by unique markers like picene (Downard et al., 2015), or coal combustion displaying a more heterogeneous distribution (Shen et al., 2013), the furniture waste matrix selectively yields a sharp, concentrated Fl and Py signature. This underscores the specific co-pyrolysis of wood lignin and synthetic binders. Such temperatures may occur during landfill fires, where oxygen access deep within the pile is limited (Wyn et al., 2020). The second group consisted of 5-ring PAHs (Bb+kF, BaF, BaP, BeP, Pe, DB), which dominated in 3 samples (TM7, TM8, TM10). Their relative content ranged between 69.9% and 71.5%. All these samples (as well as sample TM9) were collected from the centre of the landfill, where the waste fire was most intense, and the estimated temperature could have exceeded 1000 °C (Moreno & Font, 2015). The highest concentrations of 5-ring PAHs recorded in samples taken from the centre of the landfill fire are fully consistent with the mechanism of high-temperature anaerobic (anoxic) pyrolysis and indicate the specific, deep-seated nature of this process. Due to the dense packing of bulky waste, a severe oxygen deficit develops in the core of the fire while simultaneously reaching high temperatures. These conditions—as confirmed by studies on the HACA (Hydrogen-Abstraction-Carbon-Addition) mechanism and the synthesis of carbonizates and biochar–favour the secondary recombination of reactive hydrocarbon radicals into heavy, highly thermally stable 5-ring structures (Di Stasi et al., 2022; Roman et al., 2026). Furthermore, the low vapor pressure and extremely high boiling point of these compounds prevent their easy thermal distillation into the gas phase. As a result, while the lighter fractions (2–4 rings) are efficiently volatilised and carried away with the smoke into the atmosphere, the 5-ring compounds undergo selective retention and concentration in the solid matrix of the carbonized residue (Zhao et al., 2008). This observation indicates that the solid residues from the core of the furniture landfill fire act as a specific adsorbent for the heaviest, most toxic fractions of organic pollutants. The heaviest 6-ring PAHs (IP, BghiP) were detected in 12 samples in much lower concentrations than the previous ones, ranging from 0.39 to 33.857 µg/g, which accounts for 0.3% to 4%. These results are consistent with the thermodynamic conditions of PAH synthesis from the lignocellulose-polymer matrix typical of furniture waste (Orasche et al., 2012). The lower concentration of IP and BghiP in the solid fire residues therefore, results from the kinetic limitations of the waste biomass pyrolysis process, which is naturally characterised by a lower efficiency in generating macromolecular condensed structures than is the case with fossil fuels (Shen et al., 2013; Vicente et al., 2015). The 2-ring naphthalene was detected in all samples at the lowest levels, ranging from 0.001 to 0.039 µg/g. This confirms the previous assumptions regarding the remaining PAHs. Due to its low boiling point (218 °C) and high vapour pressure, naphthalene undergoes almost complete thermal desorption and immediate evacuation into the gas phase under the conditions of the high-temperature fire core (Lim et al., 2022; Vince et al., 2015). As a result, solid fire residues are naturally depleted of volatile light fractions, while simultaneously acting as a retention matrix for heavier, extremely non-volatile aromatic hydrocarbons.

### Diagnostic PAH ratios in the analysis of pollution source impacts

**Fig. 5:**
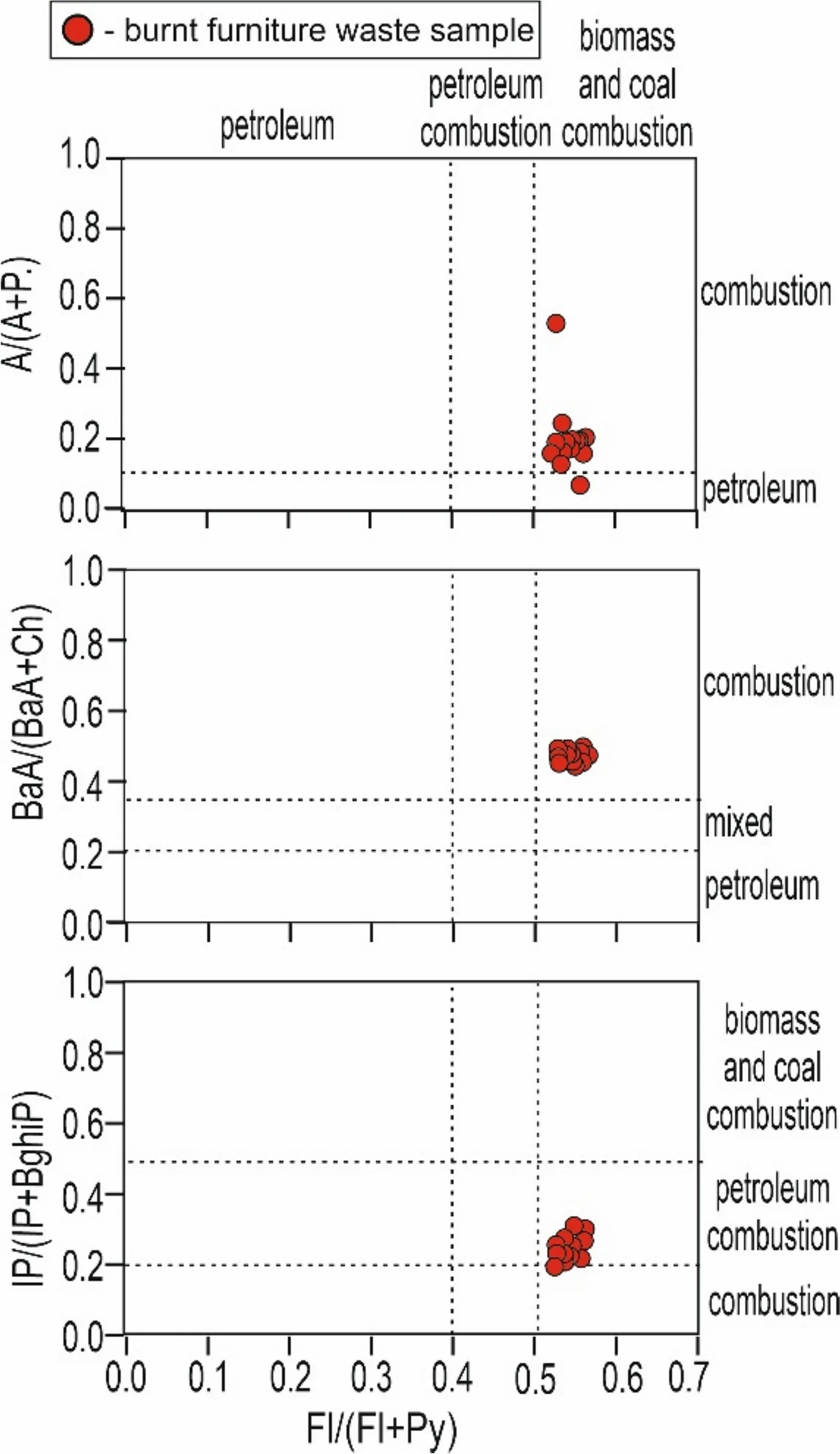
Diagnostic diagrams of PAHs burnt solid waste samples. A, anthracene, P, phenanthrene, Fl, fluoranthene, Py, pyrene, BaA, benzo(a)anthracene, Ch, chrysene, IP, indeno(1,2,3-cd)pyrene, BghiP, benzo(ghi)perylene

To determine the origin of polycyclic aromatic hydrocarbon (PAH) contamination in the investigated region, environmental studies frequently employ diagnostic ratios as a reliable source-apportionment tool (Tobiszewski & Namieśnik, 2012; Yunker et al., 2002). These molecular markers help distinguish between various anthropogenic and natural inputs, including fossil fuel combustion (Fabiańska et al., 2016), vehicular traffic, or environmental disasters (Rykała et al., 2024). For the source apportionment performed in this study (Table 2 and Fig. 5), specific isomeric pairs were evaluated, namely fluoranthene to pyrene, anthracene to phenanthrene, benzo(a)anthracene to chrysene, and indeno[1,2,3-cd]pyrene to benzo(g,h,i)perylene.

**Table 2:** Values of diagnostic PAH ratios

Sample	A/ (A+P)	Fl/ (Fl+P)	BaA/ (BaA+Ch)	IP/ (IP+BghiP)	Sample	A/ (A+P)	Fl/ (Fl+P)	BaA/ (BaA+Ch)	IP/ (IP+BghiP)
TM1	0.09	0.52	0.44	–	TM8	0.11	0.52	0.45	0.22
TM2	0.11	0.52	0.44	0.19	TM9	0.11	0.52	0.45	0.18
TM3	0.05	0.54	0.43	0.19	TM10	0.10	0.55	0.45	0.22
TM4	0.12	0.53	0.43	0.21	TM11	0.12	0.53	0.43	0.21
TM5	0.11	0.53	0.45	0.19	TM12	0.12	0.54	0.44	0.25
TM6	0.12	0.52	0.44	0.19	TM13	0.13	0.55	0.46	0.24
TM7	0.38	0.52	0.42	0.21					

P, Phenanthrene, A, Anthracene, Fl, Fluoranthene, Py, Pyrene, BaA, Benzo(a)anthracene, Ch, Chrysene, IP, Indeno(1,2,3-cd)pyrene, BghiP, Benzo(ghi)perylene, “-“, Compounds not found or present but in concentrations too low to calculate a parameter value

Analysis of the incinerated furniture waste samples revealed chemical profiles typical of biomass and hard-coal combustion, highlighting the predominant role of pyrogenic processes. This demonstrates that the fire was primarily characterized by the high-temperature, incomplete combustion of solid fuels and organic matter (pyrogenic sources), rather than from the spillage of crude oil or petroleum products (petrogenic sources) (Szramowiat-Sala et al., 2025). From a chemical perspective, since furniture consists predominantly of wood and wood-based composites such as MDF and particleboard, this plant-derived matrix undergoes rapid carbonisation when burned under oxygen-limited conditions at a landfill. The resulting pollutant profile is virtually indistinguishable from that generated during the burning or coking of hard coal. This strong resemblance stems from their shared chemical lineage; hard coal is essentially highly compressed biomass that retains the polycyclic carbon framework of its original lignin (Yunker et al., 2002).

The A/(A+P) ratio across all analysed samples ranged between 0.09–0.13 (except for sample TM7–0.38). These values hover around the critical threshold of 0.10, a threshold conventionally used in scientific literature to differentiate petrogenic inputs (< 0.10) from pyrogenic origins (> 0.10) (Tobiszewski & Namieśnik et al., 2012). This specific A/(A+P) proportion illustrates the distinct dynamics of the furniture fire; high oxygen availability accelerates the degradation of the less stable anthracene relative to the more resilient phenanthrene (Galarneau, 2008). Consequently, the combination of these low ratio values (0.09–0.13) alongside Fl/(Fl+Py) ratios exceeding 0.50 is a well-established indicator of low-temperature pyrolysis or smoldering biomass within the deeper strata of the landfill (Yunker et al., 2002).

The BaA/(BaA+Ch) ratio across all samples remained between 0.42–0.46, a range highly indicative of the thermal decomposition of solid organic matter. This interval reflects the specific nature of uncontrolled combustion of furniture waste, where the structural backbone consists primarily of natural wood and engineered wood products. Under fully developed landfill fire conditions, benzo[a]anthracene (BaA) and chrysene (Ch) are generated through high-temperature pyrosynthesis of aromatic rings derived from lignin and cellulose monomers. The observed range (0.42–0.46) matches literature data for emissions from hardwood and softwood combustion under oxygen-limited conditions, where this specific index consistently spans from 0.40 to 0.50 (Yunker et al., 2002). Furthermore, because the values did not exceed this upper threshold, it suggests that the process occurred in an open-fire environment. Under such conditions, chemical additives within the furniture—including varnishes, laminates, and polyurethane adhesives—modulate the formation rates and yields of individual PAH compounds, stabilising the final ratio at a moderately high plateau characteristic of these combustion processes (Hoffer et al., 2020; Szramowiat-Sala et al., 2025).

The IP/(IP+BghiP) ratios across the analysed samples ranged from 0.19 to 0.25. This signature appears to contradict the other diagnostic ratios, which unequivocally indicated a predominance of biomass and hard-coal combustion. However, this discrepancy may be attributed to the unique chemical composition of furniture waste, alongside the high susceptibility of these specific isomers to degradation mechanisms. Indeno[1,2,3-cd]pyrene (IP) and benzo(ghi)perylene (BghiP) are heavy, six-ring PAH compounds characterized by low vapor pressures, causing them to bind tightly to ash and particulate surfaces. Under the conditions of a rapid, open landfill fire, IP exhibits substantially lower photochemical stability and degrades more quickly than BghiP (Galarneau, 2008). This behavior causes an artificial suppression of the ratio values in post-fire samples. Furthermore, furniture waste is heavily intermixed with synthetic materials and petroleum-derived substances, including varnishes, solvents, and plastic laminates. During a fire, these components undergo intense flaming combustion, producing an emission profile enriched in BghiP, which shifts the diagnostic ratio toward values typical of liquid fuel combustion (Simoneit et al., 2005). Consequently, the IP/(IP+BghiP) range of 0.19–0.25 does not signify crude oil contamination; rather, it reflects the overlapping effects of intensive chemical additive combustion and the photochemical aging of fire-derived soot (Dvorská et al., 2011; Szramowiat-Sala et al., 2025).

The distinct chemical signature of the furniture fire is further corroborated by a comparison with historical data from other complex fires at waste disposal sites. While fires involving mixed solid waste at illegal landfills exhibit significant variability in diagnostic ratios (Fl/(Fl+Py) values ranging from 0.24 to 0.79, BaA/(BaA+Ch) from 0.24 to 0.87) and IP/(IP+BghiP) values (predominantly 0.24–0.57) (Rykała et al., 2022), also (Fl/(Fl+Py) values ranging from 0.38 to 0.74, BaA/(BaA+Ch) from 0.26 to 0.50) and IP/(IP+BghiP) values (predominantly 0.42–0.78) (Rykała et al., 2024), the furniture-derived matrix analyzed here displays a stable profile clustered within a narrow range (Fl/(Fl+Py): 0.52–0.55; BaA/(BaA+Ch): 0.42–0.46; IP/(IP+BghiP): 0.18–0.25) (Table 2). This difference can likely be attributed to the specific co-pyrolysis mechanism characteristic of treated consumer furniture.

**Fig. 6:**
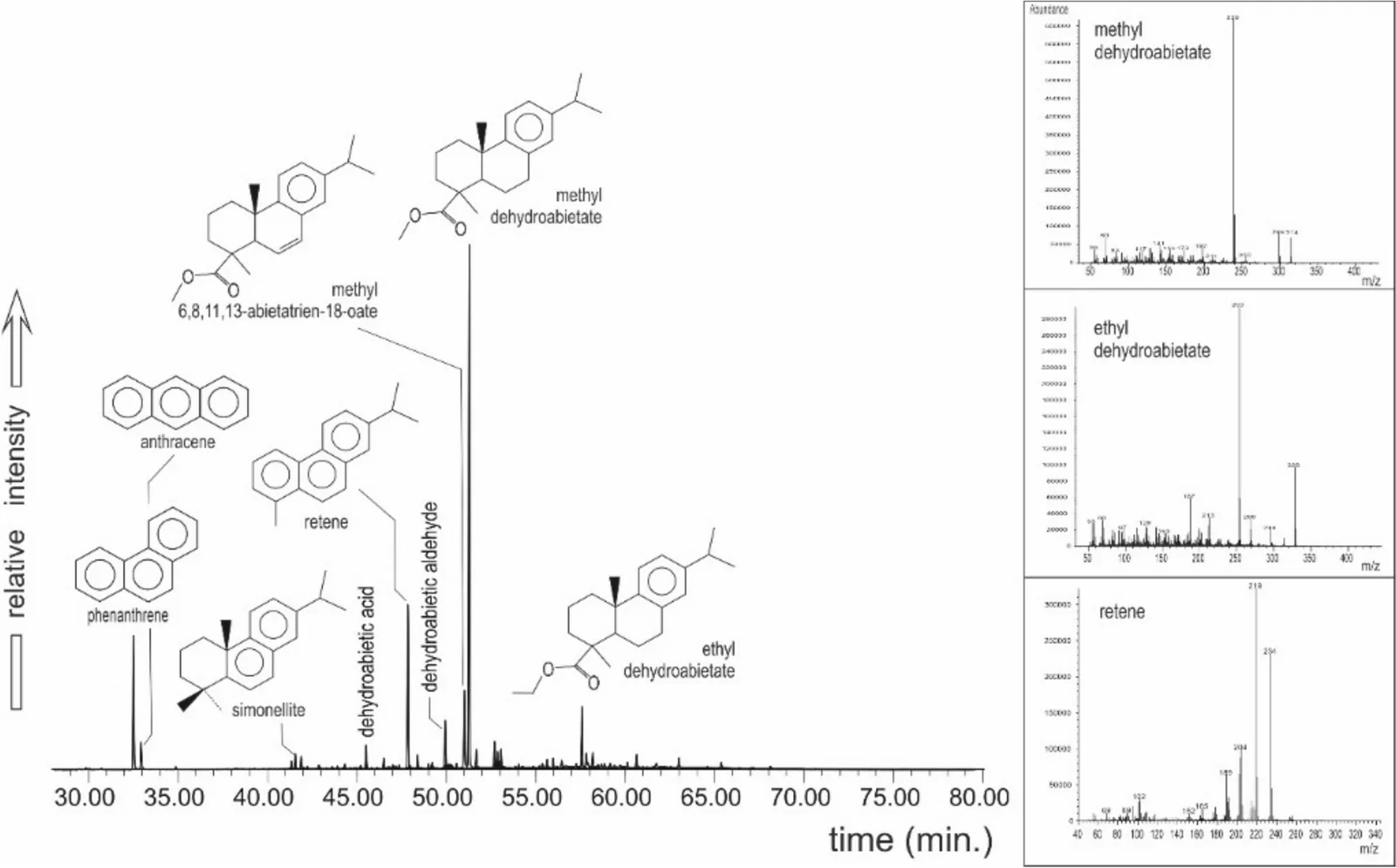
Composed chromatogram of abietane derivatives found in burnt furniture waste (*m/z* = 178, 234, 237, 284, 312, 314, 328), together with the mass spectra of selected compounds

The uniform biological nature of the furniture material is also confirmed by the presence of several abietane derivatives, e.g., dehydroabietic acid (*m/z* = 285, 300), dehydroabietic aldehyde (*m/z* = 284), and methyl and ethyl dehydroabietates (*m/z* = 314 and 328, respectively) together with their defunctionalization and aromatization products, simonellite, retene, and phenanthrene (Fig. 6). These compounds occurred found in all samples investigated. They form during coniferous wood heating, in particular, pine wood via reactions of dehydrogenation and aromatization of resin acids. Such abietane derivatives should be considered as the most important markers of burning furniture since most of inexpensive furniture are produced from pine and spruce chipboards. They are also common components of pine wood tar (Bailly et al., 2016; Davara et al., 2023; Hjulström et al., 2006).

### Alkylated PAHs in diagnostic ratios

**Fig. 7:**
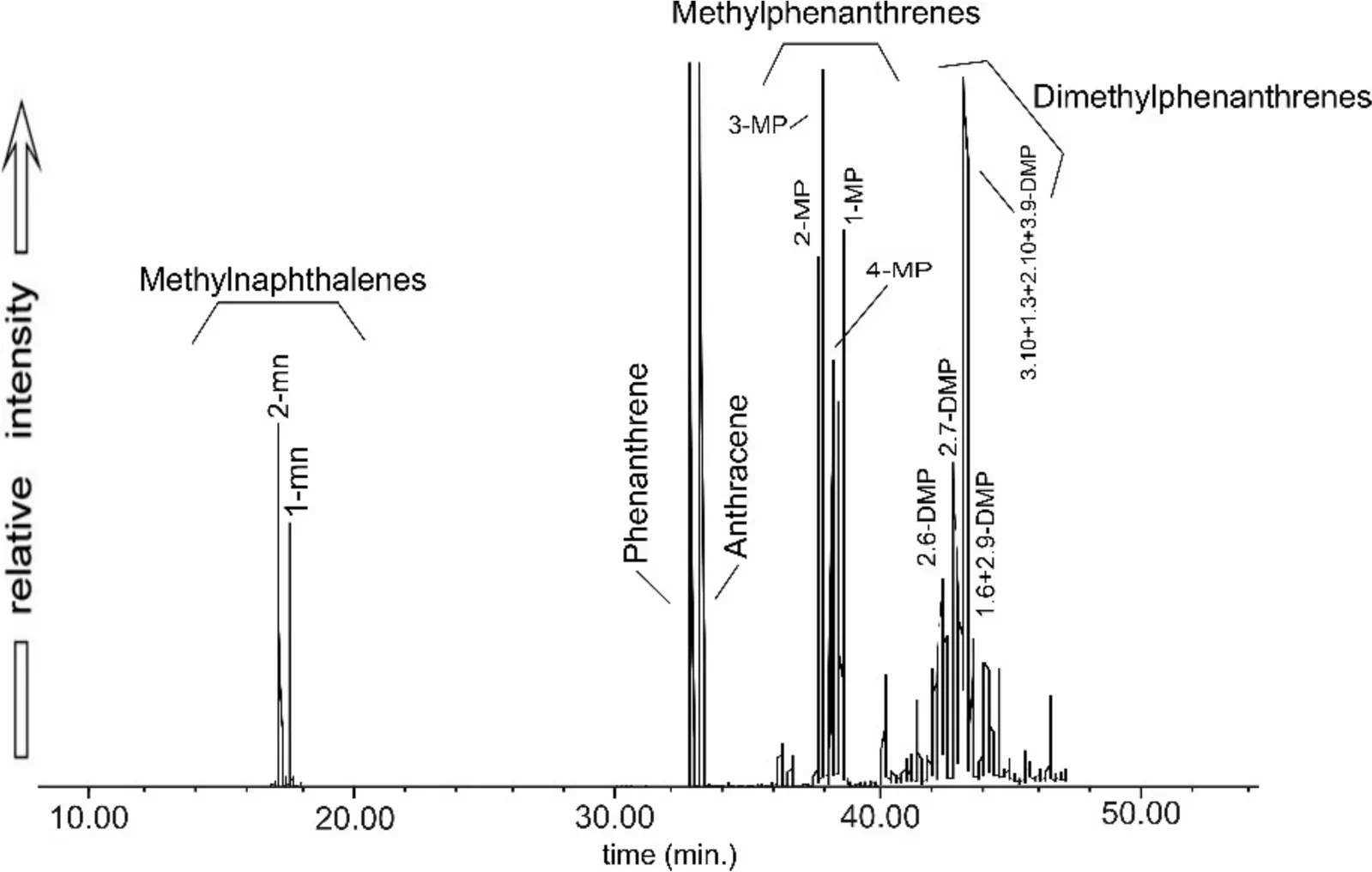
Ion chromatograms of alkylnaphthalenes (*m/z* = 142), alkylphenanthrenes (*m/z* = 192), and dimethylphenanthrenes (*m/z* = 206) in extract TM7

The analysis of the Methylnaphthalene Ratio (MNR, *m/z* = 142) (Table 3 and Fig. 7), evaluated based on the distribution of 2-methylnaphthalene to 1-methylnaphthalene (Radke et al., 1982), revealed a distinct thermal gradient between the peripheral zones and the burning core of the landfill. In the waste samples collected at a distance from the fire center, the MNR values exhibited a narrow and stable distribution ranging from 1.31 to 1.59. This consistent profile indicates exposure to more uniform, lower-temperature thermal conditions, typical of smoldering borders or areas primarily affected by smoke deposition. In stark contrast, the samples retrieved from the fire epicenter demonstrated a much wider range and higher variance, with MNR values stretching from 1.26 to 2.25. The elevation towards the maximum value of 2.25 directly marks the zones of advanced pyrolysis and intense thermal stress, where the less stable alpha-isomer (1-methylnaphthalene) underwent rapid degradation relative to the more stable beta-isomer (2-methylnaphthalene) (Yang & Mingming, 2005). Consequently, while the peripheral MNR range provides a stabilized baseline for the overall incident, the broader dispersion of 1.26–2.25 precisely delineates the highly dynamic and heterogeneous combustion processes operating within the burning core of the landfill.

**Table 3:** Values of diagnostic PAH ratios

Sample	MNR	MPI3	MPI1	DMPR	R_c_	Sample	MNR	MPI3	MPI1	DMPR	R_c_
TM1	1.36	1.02	0.30	0.97	0.58	TM8	1.36	0.84	0.35	1.30	0.61
TM2	1.59	0.73	0.36	1.33	0.62	TM9	2.25	0.82	0.31	1.12	0.59
TM3	1.58	1.43	0.20	0.78	0.52	TM10	1.26	0.93	0.33	1.05	0.60
TM4	1.23	0.75	0.34	1.34	0.61	TM11	1.43	0.73	0.41	1.52	0.65
TM5	1.40	0.89	0.35	1.23	0.61	TM12	1.36	0.68	0.43	1.64	0.66
TM6	1.29	0.73	0.40	1.48	0.64	TM13	1.31	0.78	0.37	1.54	0.62
TM7	1.41	1.15	0.32	0.66	0.59						

MNR, Methylnaphthalene ratio, MPI3, Methylphenanthrene indice, MPI1, Methylphenanthrene indice, DMPR, Dimethylphenanthrene ratio, R_c_, Reflectance equivalent

The analysis of methylphenanthrene indices (*m/z* = 192) in the waste samples collected outside the main fire zone revealed that the MPI3 values spanned a broad range from 0.68 to 1.43, whereas the samples retrieved from the burning core exhibited a noticeably narrower and lower interval between 0.82 and 1.15. A highly consistent trend was observed for the MPI1 index, which distributed across a broader range of 0.20 to 0.43 in the peripheral areas, but became strictly confined to a tight, lower interval of 0.31 to 0.35 within the fire epicenter. Under standard thermal conditions, both MPI1 and MPI3 are expected to increase with rising temperature due to the selective preservation of more stable methylphenanthrene structures (Radke et al., 1986). The paradoxically lower maximum values of MPI3 (1.15) and the strict suppression of MPI1 (0.31–0.35) inside the burning core reflect the distinct dynamics of an open-system furniture waste landfill fire operating under high temperatures. However, with the increasing thermal stress also more temperature-resistant methylphenanthrene isomers will begin to decay. That leads to both ratios decreasing in value. At this extreme thermal threshold, the rapid destruction and dealkylation of the methylphenanthrene fraction outpace any structural shifts, preventing the indices from reaching higher values (Zheng et al., 2023). Conversely, the broader ranges observed in the peripheral zones (up to 1.43 for MPI3 and 0.43 for MPI1) indicate that the lower-temperature smoldering regimes on the outskirts allowed these volatile compounds to survive and undergo progressive thermal transformation without facing immediate molecular destruction (Fabiańska et al., 2013; Simoneit, 2002).

The Dimethylphenanthrene Ratio (DMPR, *m/z* = 206) further reinforces this spatial trend, yielding a broader and higher range of 0.78 to 1.48 in the peripheral zones compared to a narrower, downward-shifted interval of 0.66 to 1.30 within the fire epicenter. This consistent compression of the dimethylated fraction under extreme core temperatures provides independent molecular confirmation that advanced thermal dealkylation consistently outpaces isomerization pathways throughout the burning landfill horizon (Li et al., 2024; Radke, 1986). Within the peripheral areas, the retention of values up to 1.48 demonstrates that the lesser thermal gradient allowed the more structurally complex dimethylated isomers to accumulate steadily without facing structural breakdown. Conversely, the strict capping at 1.30 in the burning core confirms that even these highly resilient multi-methylated frameworks face severe structural destabilization when exposed to the high intensity of the landfill fire (Lefort & Tsang, 2011).

### *Calculated reflectance (R*_*c*_*) as a solid-phase thermal proxy*

To independently validate the localized intensity of the solid waste landfill fire, the calculated vitrinite reflectance equivalent (R_c_) was employed as a solid-phase petrographic proxy. During the high-intensity combustion of manufactured furniture waste, the cell-wall components of wood fractions—primarily lignin and cellulose—undergo rapid carbonization, forming technogenic char particles that structurally mimic natural macerals (Misz-Kennan & Fabiańska, 2011). Unlike volatile and extractable polycyclic aromatic hydrocarbons, which suffer destructive alteration and isomerization locking at elevated temperatures, the structural ordering and reflectance of the residual carbon framework evolve progressively and irreversibly with increasing thermal stress (Burnham & Sweeney, 1989; Nádudvari & Fabiańska, 2016). Consequently, assessing the optical reflectance properties of these charred furniture matrices provides a highly reliable, cumulative record of the peak heating regimes across the different landfill sectors.

The experimental results for the calculated reflectance equivalent (R_c_) revealed a highly uniform profile across the entire study area, demonstrating a narrow range of 0.58 to 0.66 in the peripheral zones and an even tighter clustering of 0.59 to 0.61 within the fire epicenter (Table 4). In traditional organic petrology, these stable values around 0.60% typically reflect moderate thermal maturation under steady-state conditions (Radke et al., 1986). The absence of an elevated R_c_ signature in the core samples (0.59–0.61), where molecular markers unequivocally indicated combustion in high temperatures, points to a distinct kinetic phenomenon characteristic of open-system landfill fires (Courty et al., 2020). Under extreme and rapid flash pyrolytic conditions, the organic components of the furniture waste undergo instantaneous volatilization and destructive ash conversion rather than progressive structural graphitization. Because the optical reflectance of char matrices requires sustained thermal exposure to undergo aromatic ring condensation and structural ordering, the intense but transient thermal shock of the fire core effectively arrested the optical evolution of the vitrinite-like residues at this baseline stage (Cetin et al., 2004). Conversely, the peripheral zones, characterized by prolonged low-temperature smoldering and extended exposure times, allowed the technogenic char to achieve a fully comparable and stabilized structural maturity of up to 0.66 without facing direct molecular oxidation (Fabiańska et al., 2013).

**Table 4:** Organic phosphate concentrations in the Tychy samples (µg/g), (mg/g)

Sample	TCiPP (µg/g)	TPhP (mg/g)	TCP (mg/g)	Sample	TCiPP (µg/g)	TPhP (mg/g)	TCP (mg/g)
TM1	0.44	7.98	0.86	TM8	2.25	12.26	236.81
TM2	2.08	10.53	3.82	TM9	10.29	3.24	1.07
TM3	3.71	12.02	2.49	TM10	7.71	10.04	4.77
TM4	2.83	9.45	1.27	TM11	2.20	43.97	1.50
TM5	0.82	5.66	3.46	TM12	1.19	13.13	35.98
TM6	2.87	14.32	3.32	TM13	1.87	22.57	18.97
TM7	10.63	14.88	0.97				

TCiPP, tris(1-chloro-2-propyl), TPhP, triphenyl phosphate, TCP, tricresyl phosphate

### Distribution of organophosphate esters (OPEs) in incinerated waste samples

Three organophosphate esters were detected in all investigated samples: tris(1-chloro-2-propyl) phosphate (TCiPP), triphenyl phosphate (TPhP), and tricresyl phosphate (TCP) (Table 4). The highest concentrations of TCiPP, exceeding 5 µg/g, were detected in samples TM7, TM9, and TM10 (10.63, 10.28, and 7.7 µg/g, respectively). TPhP exhibited high concentrations across all analysed samples; concentrations up to 10 mg/g were found in four samples (TM1, TM4, TM5, TM9), whereas in the remaining nine samples, values reached up to 43.97 mg/g (TM11). Significant amounts of TCP were detected in three samples (TM8, TM12, TM13), while in the remaining samples, its levels ranged from 0.65 to 4.77 mg/g.

The presence of these three compounds provides clear evidence that municipal waste, including furniture, was incinerated at the landfill site, rather than clean wood or vegetation (Wensing et al., 2005). The remarkably high concentration of TPhP is attributed to its widespread use as a plasticiser in varnishes, polishes, and wood adhesives (Wei et al., 2015). Conversely, the chlorinated compound TCiPP is a typical flame retardant. During a fire, the majority of this compound most likely volatilises rapidly into the air, leaving only minor trace amounts in the ash (Wensing et al., 2005). TCP serves as a primary plasticising agent, for instance, in plastic veneers (Eede et al., 2011). The detection of such significant quantities of all three organophosphate esters demonstrates that these compounds exhibit high thermal stability and can withstand intense fire conditions, and become permanently bound to the resulting ash and soot (Li et al., 2019). Since these compounds play a critical role in formulae of furniture varnishes, binders, and protective coatings they can be considered markers of contamination derived from burnt furniture.

The high concentrations of organophosphate esters (OPEs) detected in the ash and soot pose a significant long-term threat in terms of uncontrolled leachate formation from the fire site. The intense fire at the furniture waste landfill led to the thermal degradation of polymers, releasing entrapped plasticisers and flame retardants. Due to their specific physicochemical properties, these compounds readily migrate into the liquid phase upon contact with rainwater or firefighting water (Fabiańska et al., 2020; Rykała et al., 2023). necessitating the immediate containment and monitoring of leachate at the fire site to protect local aquatic ecosystems (Rykała et al., 2026).

### Diagnostic OPE profiles for landfill furniture waste source identification

The presence of TCiPP, TPhP, and TCP in all the tested samples clearly proves that furniture waste was the main source of pollution. While standard polycyclic aromatic hydrocarbons (PAHs) are produced during almost any fire, these specific organophosphate esters are artificial additives found only in manufactured goods (Blum et al., 2019). In the furniture industry, TCiPP is commonly used as a flame retardant in soft upholstery foams, TPhP is added to plastics, and TCP is used to make wood lacquers and fabric coatings more durable (Gao et al., 2016; Liu et al., 2016). The fact that these three specific chemicals survived the fire confirms that the landfill materials consisted of treated consumer furniture rather than typical municipal waste (Rykała et al., 2023). This specific OPE profile distinctly differs from other fire scenarios. In typical municipal solid waste (MSW) landfill, chlorinated retardants prevail in much lower ranges (Wei et al., 2015). In contrast, the extreme dominance of TPhP (up to 43.97 mg/g) along with TCP and TCiPP is a unique feature of burnt furniture waste, directly reflecting the intensive industrial application of treated foams, protective coatings, and wood lacquers (Liu et al., 2016). Additionally, the widespread detection of these compounds highlights their role as stable environmental tracers that remain in the waste matrix even after facing intense heat. This means that monitoring these organic phosphates provides crucial information about what types of industrial materials were burned, helping emergency teams choose the right safety measures. Therefore, tracking this specific organic phosphate mix gives researchers a very precise tool to identify furniture waste fires and separate them from other types of environmental pollution.

### Health risk analysis indicators

In studies on landfill fires and other uncontrolled emissions (e.g., fires involving tires, plastics, or fuels), mutagenicity and carcinogenicity indices are key toxicological tools. They enable the assessment of how toxic the released smoke plume or resulting ash is to human health and the environment (DeMarini & Linak, 2022). The mutagenicity equivalent (MEQ) index in the analysed samples ranged from 1.59 to 5.78 for those collected outside the center of the landfill fire. Samples from the epicentre exhibited the highest values (exceeding 100) (Table 5). This indicates the highest concentrations at the core of the fire site. Since these heaviest, most genotoxic compounds are characterised by low volatility and high affinity for carbon matrices, they underwent strong, localised concentration in the ash and soot at the immediate site of the fire. In contrast, the much lower MEQ values detected outside the centre merely reflect the extent of secondary, dispersed deposition of fine fire aerosol particles, demonstrating a several-hundred-fold decrease in mutagenic potential with increasing distance from the point emission source (Odekanle et al., 2024). The MEQ results are consistent with previous studies concerning solid waste fires (Pająk & Reindl, 2026; Rykała et al., 2022). The carcinogenicity equivalent (TCDD-TEQ) also showed lower concentrations, ranging from 0.01 to 0.04, in samples collected outside the epicenter of the fire. However, this still represents an elevated concentration (Wan et al., 2023) in the sampled solid waste, resulting from the consequences of the investigated furniture waste fire. The toxicity index ΣPAH_carc_/ΣPAH in most of the analysed burnt furniture waste samples remained below 0.37 (except for TM7, TM8, and TM10), indicating a relatively low risk to human health from carcinogenic PAHs, for which the maximum risk factor is 1 (Rogula-Kozłowska et al., 2013). Nevertheless, it should be noted that this assessment changes in the context of chronic exposure. In the case of long-term human exposure (defined in classic toxicological models as a period exceeding 7 years), even moderate concentrations of the ΣPAH_carc_ fraction may lead to secondary bioaccumulation of toxins and the induction of a genotoxic effect. Consequently, over a long-term perspective, the analysed waste matrices—with particular emphasis on samples TM7, TM8, and TM10–may pose a potential threat to human health (Cheng et al., 2023). The total Σ16PAHs [µg/g] in the analysed samples also confirms the existence of a pollution source: the landfill fire.

**Table 5:** Indicators of environmental and health toxicity

Sample	MEQ	TCDD-TEQ	BaPE	RTBaP	ΣPAH_carc_ /ΣPAHs	Σ16PAHs (µg/g)	Sample	MEQ	TCDD-TEQ	BaPE	RTBaP	ΣPAH_carc_/ΣPAHs	Σ16PAHs (µg/g)
TM1	1.59	0.01	1.03	1.25	0.27	33.12	TM8	45.06	0.32	12.53	655.51	0.84	203.21
TM2	3.26	0.02	2.11	2.55	0.37	43.94	TM9	3.54	0.02	2.25	2.73	0.35	45.24
TM3	5.78	0.04	3.73	4.43	0.38	59.91	TM10	112.61	0.78	30.05	1684.36	0.74	615.48
TM4	3.11	0.02	1.98	2.50	0.23	73.86	TM11	1.68	0.01	1.08	1.33	0.28	31.85
TM5	5.48	0.04	3.47	4.22	0.35	80.78	TM12	3.55	0.03	2.28	3.02	0.19	135.80
TM6	2.17	0.02	1.23	1.64	0.26	46.38	TM13	4.55	0.03	2.92	3.72	0.22	120.81
TM7	628.89	4.35	155.31	9873.98	0.82	2960.72							

MEQ, Mutagenicity equivalent, TCDD-TEQ, Carcinogenicity equivalent, BaPE, Benzo(a)pyrene equivalent, RTBaP, Toxicity equivalent

### Screening-level environmental and human-health risk assessment

**Fig. 8:**
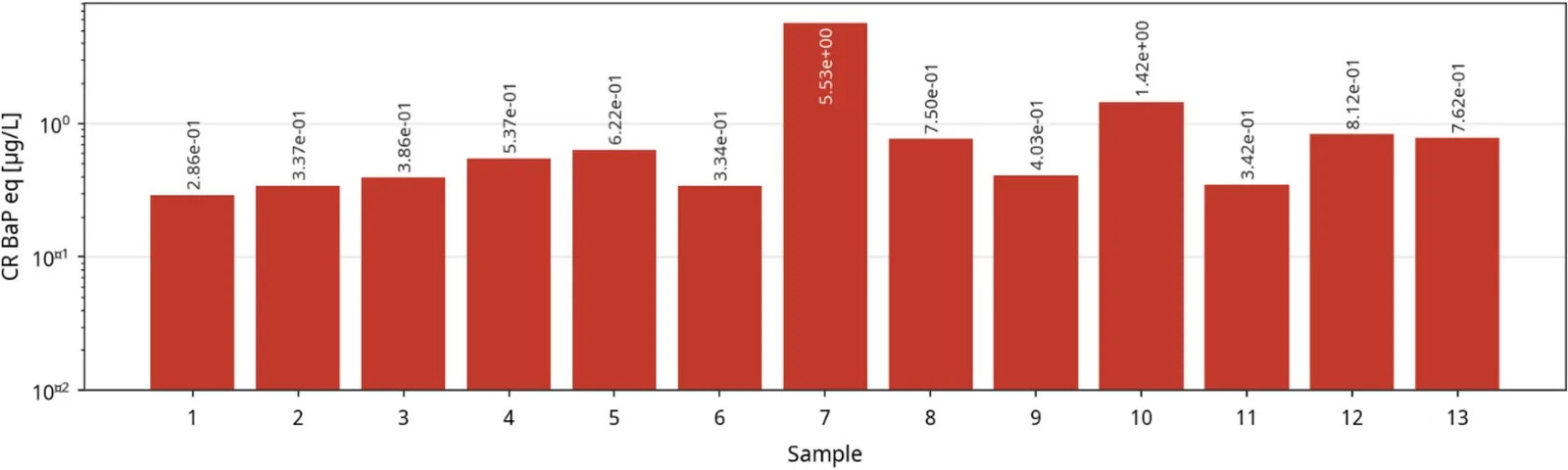
Estimated concentrations of benzo(a)pyrene equivalents (BaPeq) in groundwater compared to the regulatory limit

Under the equilibrium, no-attenuation screening scenario, estimated source-zone pore-water BaP-equivalent concentrations ranged from 0.24 µg/L (TM1) to 5.53 µg/L (TM7). These are not measured aquifer groundwater concentrations. BaP-equivalent concentrations were used only in the mixture cancer-risk calculation; a regulatory benchmark for benzo(a)pyrene should be compared only with the modelled concentration of benzo(a)pyrene (Fig. 8).

For the hypothetical water-ingestion screening scenario, calculated cancer-risk values ranged from 2.78×10⁻^6^ to 6.50×10⁻^5^. These values are conditional on the equilibrium and no-attenuation assumptions and should not be interpreted as measured exposure doses or evidence of groundwater regulatory non-compliance. The highest screening estimate was observed for TM7 (CR = 6.50×10⁻^5^).

For the same hypothetical water-ingestion screening scenario, non-carcinogenic HI values were below 1.0, ranging from 0.025 (TM11) to 0.370 (TM7). This finding applies only to the stated screening assumptions and requires confirmation with measured leachate and groundwater data.

**Fig. 9:**
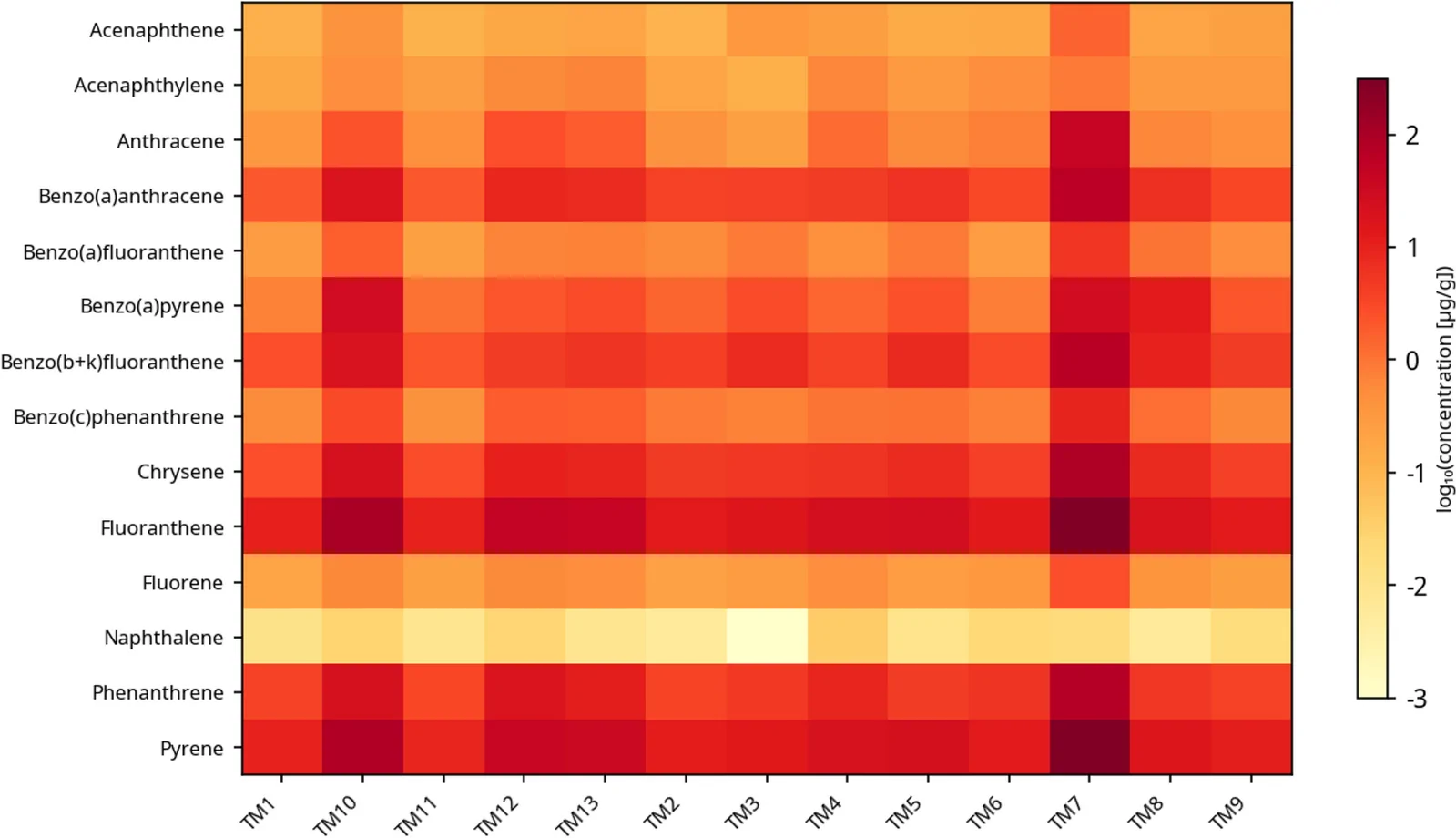
Heatmap illustrating the contribution of individual PAHs to the total Carcinogenic Risk (CR)

A detailed analysis of the risk drivers revealed that carcinogenic risk is predominantly governed by a few high-molecular-weight PAHs. As illustrated in the heatmap (Fig. 9), benzo(a)pyrene, benzo(b+k)fluoranthene, benzo(a)anthracene and fluoranthene are the primary contributors to the total CR across all samples. For instance, in the most contaminated sample (TM7), benzo(a)pyrene alone accounted for approximately 43% of the total carcinogenic risk (CR_BaP = 2.78E^−5^), despite its relatively low mobility (Koc = 1,995,262 L/kg). The average share of fluoranthene in CR is 76.98% in all samples. This is due to the combination of high concentration in the waste, relatively low Koc (97,724 L/kg, which indicates higher mobility), and a significant RPF = 0.08.

Within the water-phase screening model, benzo(a)pyrene alone is not a sufficient indicator of the mixture risk because its high hydrophobicity limits its estimated release to water. This does not justify comparison of a BaP-equivalent mixture concentration directly with a compound-specific benzo(a)pyrene regulatory limit.

Fluoranthene was an important contributor to the calculated mixture risk because of its high source concentration, relatively lower Koc and assigned RPF.

**Fig. 10:**
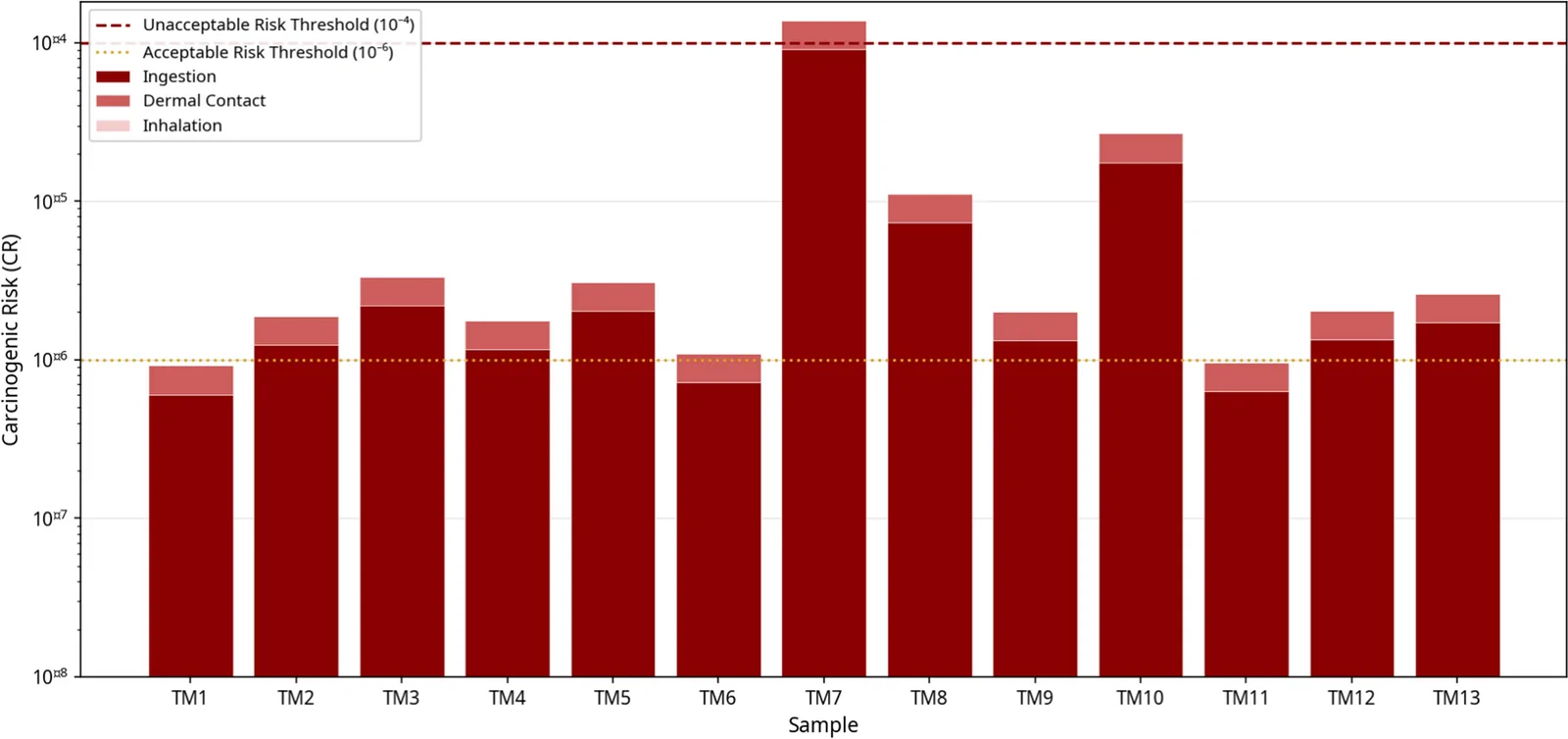
Carcinogenic Risk (CR) by exposure pathway

The direct-contact cancer-risk results are highly dependent on the assumed incidental soil/dust ingestion rate. Under the upper-bound 100 mg/day soil-plus-dust scenario reported in Table 6, total CR ranged from 9.20×10⁻^7^ (TM12) to 1.39×10⁻^4^ (TM7), and TM7 exceeded 1.0×10⁻^4^. When only IRs was varied while the other pathway contributions were held constant, the corresponding TM7 estimate was approximately 9.34×10⁻^5^ at 50 mg/day and 5.69×10⁻^5^ at 10 mg/day (Fig. 10). Thus, the direct-contact results identify TM7 as the sample of greatest concern, but the risk category should be interpreted as a conservative screening result rather than an estimate of an actual exposure dose. The carbonized residue should nevertheless be handled with appropriate personal protective equipment during site-reclamation work.

**Table 6:** Summary of the analysis of soils results

Sample	BaP (µg/g)	CR (ingestion)	CR (dermal)	CR (total)	Sample	BaP (µg/g)	CR (ingestion)	CR (dermal)	CR (total)
TM1	1.031	6.05e−07	3.14e−07	9.20e−07	TM8	12.534	7.36e−06	3.82e−06	1.12e−05
TM2	2.107	1.24e−06	6.42e−07	1.88e−06	TM9	2.249	1.32e−06	6.85e−07	2.01e−06
TM3	3.731	2.19e−06	1.14e−06	3.33e−06	TM10	30.045	1.76e−05	9.15e−06	2.68e−05
TM4	1.984	1.16e−06	6.04e−07	1.77e−06	TM11	1.083	6.36e−07	3.30e−07	9.66e−07
TM5	3.471	2.04e−06	1.06e−06	3.10e−06	TM12	2.278	1.34e−06	6.94e−07	2.03e−06
TM6	1.226	7.20e−07	3.73e−07	1.09e−06	TM13	2.917	1.71e−06	8.88e−07	2.60e−06
TM7	155.310	9.12e−05	4.73e−05	1.39e−04

Under the upper-bound 100 mg/day scenario, incidental soil/dust ingestion contributed approximately 77% of total CR, dermal contact approximately 23%, and inhalation of resuspended dust less than 0.01%. This distribution is driven by the selected exposure parameters and PAH-specific absorption factors; PAH bioavailability/bioaccessibility in the carbonized matrix was not measured.

The direct-contact model assumes incidental contact with soil/dust derived from charred waste, although neither contact frequency nor PAH bioaccessibility in the carbonized matrix was measured. The water-phase model assumes local solid–water equilibrium and no attenuation between the source and groundwater. Waste heterogeneity, infiltration, desorption kinetics, dissolved organic matter, sorption during transport, dilution and degradation may cause actual leachate and groundwater concentrations to differ from the calculated screening values. Accordingly, these estimates identify conditions that warrant monitoring and management; they do not quantify an actual individual dose or establish groundwater regulatory non-compliance.

**Fig. 11:**
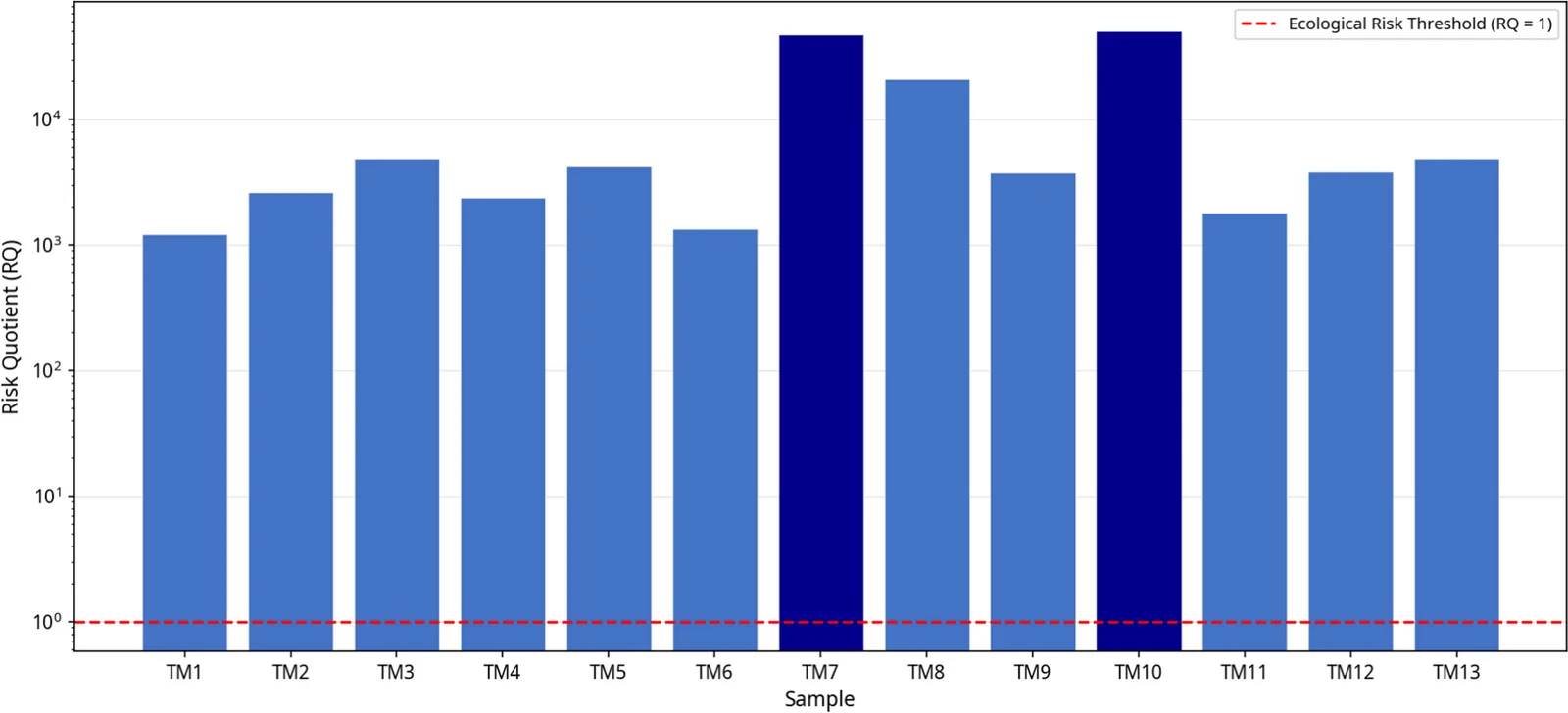
Ecological Risk Quotient for benzo(a)pyrene in solid waste samples

The calculated RQ values for BaP in the analysed solid waste samples were exceptionally high, ranging from 1206 (TM12) to 47,309 (TM7). Since an RQ > 1 indicates a high ecological risk, all 13 samples exhibited extreme ecological hazard, with RQ values exceeding the ecological risk threshold by three to five orders of magnitude (Fig. 11). Even the least contaminated sample (TM12, BaP = 0.79 µg/g) exceeded the PNEC by a factor of 2403, while the most contaminated sample (TM7, BaP = 28.39 µg/g) exceeded it by a factor of 47,309. Furthermore, all samples significantly exceeded the Polish regulatory soil quality standards for both Group I (0.2 mg/kg) and Group III (2.0 mg/kg) land-use categories, confirming the severe contamination of the solid waste material.

These findings indicate that the solid furniture waste deposited at the Tychy site poses a severe and immediate ecological risk to soil biota, including invertebrates, microorganisms, and plants. The persistence and bioaccumulation potential of high-molecular-weight PAHs such as BaP, combined with the extreme concentrations measured in the samples, suggest that uncontrolled leaching and dispersion of these contaminants may lead to long-term degradation of the local ecosystem. Immediate remediation measures and containment of the waste material are therefore warranted to prevent further ecological damage.

A comparison of the soil and groundwater risk assessments reveals fundamentally different risk profiles for the two environmental compartments. The soil HHRA indicates that direct contact with the contaminated solid waste poses an unacceptable carcinogenic risk (CR > 10⁻^4^) for the most contaminated sample (TM7), and an elevated risk (CR > 10⁻^6^) for all samples. In contrast, the groundwater HHRA, based on the leaching model, yielded CR values in the range of 2.78×10⁻^6^ to 6.50×10⁻^5^–elevated but below the unacceptable threshold. Direct soil contact involves undiluted PAH concentrations, while groundwater exposure involves concentrations that are substantially attenuated by the partitioning process governed by the organic carbon–water partition coefficient (Koc).

The key risk driver also differs between the two pathways. In the groundwater scenario, fluoranthene emerged as the primary risk driver due to its relatively high mobility and significant RPF. In the direct soil exposure scenario, benzo(a)pyrene and other high-molecular-weight PAHs with high RPF values dominate the risk, as their mobility limitations are irrelevant in the context of direct contact.

## Conclusions

The results of this study demonstrate that an uncontrolled fire at a furniture waste landfill can constitute a qualitatively distinct contamination event, fundamentally different from passive leaching under normal landfill conditions. Rather than a gradual release of pollutants, the fire acted as a high-intensity pyrolytic reactor, selectively synthesizing and concentrating the heaviest, most toxic PAH fractions—particularly 5-ring compounds—within the solid residues at the fire's core. This mechanism of in situ thermal enrichment means that the post-fire ash represents a far more hazardous secondary source of contamination than the original, unburned waste. The simultaneous thermal mobilization and subsequent ash-binding of organophosphate flame retardants (TCiPP, TPhP, TCP) further compounds this hazard, creating a chemically complex leachate precursor that is highly susceptible to mobilization by rainwater or firefighting water.

The diagnostic parent PAH ratios—specifically the A/(A+P), BaA/(BaA+Ch), and IP/(IP+BghiP) profiles–collectively outline a complex open-system fire regime dominated by heterogeneous combustion conditions, active biomass smoldering within deeper waste strata, and distinct photochemical aging of the fire-derived soot. These pyrogenic source indicators reflect the hybrid composition of landfilled furniture waste, where chemical additive combustion and rapid oxidative degradation lines alter traditional emission profiles without signifying independent petroleum contamination. Additionally, the persistent detection of specific abietane derivatives–including dehydroabietic acid, dehydroabietic aldehyde, and methyl and ethyl dehydroabietates–unequivocally verified the presence of coniferous softwood matrices within the burned substrate. The survival of these diterpenoid biomarkers confirms that natural pine resin components undergo stable thermal transformation into wood tar during open landfill fires, providing a valuable botanical baseline that complements synthetic chemical markers for full waste source reconstruction.

Several compound groups investigated in this project can act as markers of contamination derived from burnt furniture, present in soil, air particulate matter and water. In particular, co-occurrence of organic phosphates and a set of aromatized abietane derivatives testify to this origin.

The structural analysis of alkylated polycyclic aromatic hydrocarbons (PAHs) provided a highly resilient, cross-validated chemical fingerprint of the furniture waste fire incident. Distinct spatial thermal gradients were successfully mapped using the Methylnaphthalene Ratio, which demonstrated structured stability in the peripheral zones but marked high-temperature variance in the burning core. Furthermore, the paradox of suppressed methylphenanthrene and dimethylphenanthrene indices within the epicenter empirically confirms that advanced thermal dealkylation and volatile cracking entirely outpaced steady structural isomerization pathways under open-system combustion regimes exceeding the critical temperature threshold. Conversely, the wider index ranges preserved in the peripheral technogenic horizons document lower-temperature smoldering and extended exposure times, which allowed structural transformations to proceed without facing immediate molecular degradation.

The risk assessment framework applied in this study reveals a critical asymmetry between exposure pathways that has direct implications for site management and regulatory monitoring. While direct soil contact with the contaminated residues poses an unacceptable carcinogenic risk driven primarily by high-molecular-weight PAHs such as benzo(a)pyrene, the groundwater risk profile is governed by an entirely different compound – fluoranthene—owing to its substantially greater mobility in the soil–water system. This finding challenges the conventional reliance on benzo(a)pyrene as the sole indicator compound in post-fire groundwater monitoring systems and underscores the need for a multi-compound approach. The extreme ecological risk quotients calculated for all samples further indicate that the contaminated residues pose an immediate threat to soil biota well beyond any regulatory threshold, reinforcing the urgency of physical containment and remediation.

## Data Availability

No datasets were generated or analysed during the current study.
